# Quadripartite bond length rule applied to two prototypical aromatic and antiaromatic molecules

**DOI:** 10.1007/s00894-023-05498-4

**Published:** 2023-03-13

**Authors:** Łukasz Wolański, Wojciech Grochala

**Affiliations:** Centre of New Technologies, University of Warsaw, S. Banacha 2C, 02-097 Warsaw, Poland

**Keywords:** Aromaticity, Antiaromaticity, Benzene, Cyclobutadiene, Molecular orbital theory

## Abstract

**Context:**

In 2000, a remarkably simple relationship was introduced, which connected the calculated geometries of isomolecular states of three different multiplicities. These encompass a ground single state, the first excited triplet state, as well as related radical anion and radical cation. The rule allows the prediction of the geometry of one of the species if the three remaining ones are known. Here, we verify the applicability of this bond length rule for two small planar cyclic organic molecules, i.e., benzene and cyclobutadiene, which stand as prototypical examples of, respectively, aromatic and antiaromatic systems. We see that the rule works fairly well to benzene, and it works independently for quinoid as well as for anti-quinoid minima, despite the fact that radical anion species poses challenges for correct theoretical description.

**Methods:**

To obtain chosen electronic state equilibrium geometries, three types of computational approaches were utilized: fast and efficient density functional theory DFT, the coupled cluster method CC2, the complete active space self-consistent field (CASSCF) approach, and the most accurate but also resource-consuming perturbation theory with multireference wavefunction (CASPT2) with a default value and without IPEA-shift. Dunning and co-workers correlation-consistent basis sets (aug-)cc-pVXZ (X = D, T, Q) were employed. Gaussian 16 revision A.03, Turbomole 7.1, and Molcas 8.0 computational software were used.

**Supplementary information:**

The online version contains supplementary material available at 10.1007/s00894-023-05498-4.

## Introduction

Geometry optimization as a multi-step process is usually the longest part of the typical quantum chemical computations. For that reason, geometry optimization is often carried out using a less precise method than the final technique used for determining other properties. Alternatively, a pre-optimization may be carried out at some low level of theory, followed by a more rigorous one, particularly for large molecules. Geometry optimization is also a process that requires some chemical knowledge and intuition, since initial atom positions have to be defined first. An unrealistic choice of a starting geometry can lead to their convergence to the structures corresponding to saddle points and/or calculations may take an impractically long time. This is particularly important when it concerns the optimization of molecules in their excited electronic states or of the even-electron (free radical) systems. In principle, this additionally requires the use of more resource-consuming methods (i.e., CC2 vs. MP2, TDDFT vs. DFT, with rather multi- than single-configurational wavefunction) than in the case of the ground-state equilibrium structures.

For these reasons, it is highly desirable to be able to propose a starting structure for optimization which is a result of an educated guess and as such could lead to much faster convergence. About 2 decades ago, Grochala, Albrecht, and Hoffmann have observed a remarkably simple relationship (subsequently labeled by Parr and Ayers as “GAH rule”), i.e., the corresponding bond lengths in the cationic (R^+^), anionic (R^‒^), and neutral (R^0^) systems (all in their electronic ground states) together with neutral structure in its first triplet excited state (R^0^_T1_) approximately satisfy the equation (Eq. 1):

1 $$\mathrm{\Delta GAH}\left(\mathrm{R}\right)={R}^{+}+{R}^{-}-{R}_{T1}^{0}-{R}^{0}\approx 0$$

This approximate relationship was proposed based on rather low-level (at least by today’s standards) quantum mechanical calculations for a handful of inorganic (largely diatomic) and organic molecules, both neutral, cationic and anionic, among those C_2_, C_2_H_2_, C_2_H_4_, C_2_H_6_, N_2_H_2_, B_2_H_2_, CO, CN^‒^, N_2_, NO^+^, and three more complex hydrocarbons. These authors have noted that the relationship expressed by Eq. 1 seems to be most accurate, if the ground state molecule is nondegenerate and equilibrium geometries of all structures ale reasonable similar [1]. Moreover, it applies exclusively to bonds constituting the chromophore part of a molecule and works best for systems with conjugated double bonds.

While computational effort related to verifying the validity of the GAH rule was rather limited, the reasons behind its seeming success are far from being obvious. Indeed, the quest for the rule was inspired by a simplistic molecular orbital (MO) picture and perturbation theory in its most simple implementation, which may obviously be of a great didactic value. I.e., if one uses a one-orbital basis set for each atom in a diatomic, a classical two-MO picture of electronic structure emerges, with the bonding highest occupied molecular orbital (HOMO) and antibonding lowest unoccupied molecular orbital (LUMO) orbital. The two-electron singlet ground state has HOMO doubly occupied and an empty LUMO, so it maximizes the bonding between both atoms. A subtraction of one electron (to form a radical cation) may be treated as a perturbation, due to which bonding strength decreases and the interatomic bond elongates. An addition of one electron (to form a radical anion) is yet another perturbation, due to which the antibonding effect appears and the bond weakens and elongates again. Usually, the bond weakening associated with the removal of one electron from HOMO is slightly weaker than the one related to the occupation of LUMO by one electron, and that is because “the antibonding orbital is more antibonding than the bonding orbital is bonding.” Nevertheless, the formation of an excited triplet state from the ground state singlet is associated with *both* effects in the same time, i.e., with two effectively antibonding effects due to decreased occupancy of HOMO and increased one of LUMO simultaneously. Therefore, it is not totally unexpected that the bond weakening is now more-less a sum of the two effects seen for two distinct free radical species.

Based on the MO theory in its two-center two-orbital implementation, one may additionally deduce that the GAH rule should work best for electronic manifold made up of π type orbitals rather than σ ones. This is because the first excited triplet state of a σ bond corresponds usually to a fully dissociated bond, and thus the effects of the singlet → triplet excitation for molecular geometry are so large that such excitation cannot be treated as a small perturbation of a system. This naturally explains why the applicability of the rule was documented before for systems with double or triple bonds, either isolated or conjugated. It is also easy to understand why the rule finds most applicability for the chromophore part of a molecule; note that a bond very distant from a chromophore and not conjugated with it via a π system does not experience any major bond length changes upon one-electron perturbations within the chromophore (i.e., for such distant bond, R^+^  = R^‒^ = R^0^ = R^0^_T1_) and therefore the relationship expressed by Eq. 1 still holds but it becomes trivial.

While the MO theory served as the initial inspiration of the rule, its applicability to diverse molecular systems is far from ideal, especially when strong electronic correlation effects apply. In a separate line of reasoning, Ayers and Parr managed to show how the Fukui function can rationalize this rule by noticing that for nondegenerate ground states it is advantageous to have a large band gap—i.e. chemically hard systems should best follow the rule [2]. Following that, an extension of “GAH approximation” was proposed by Morell and co-workers as a way of calculating the potential energy profile of the reaction in its first electronic excited state [3].

**Fig. 1:**
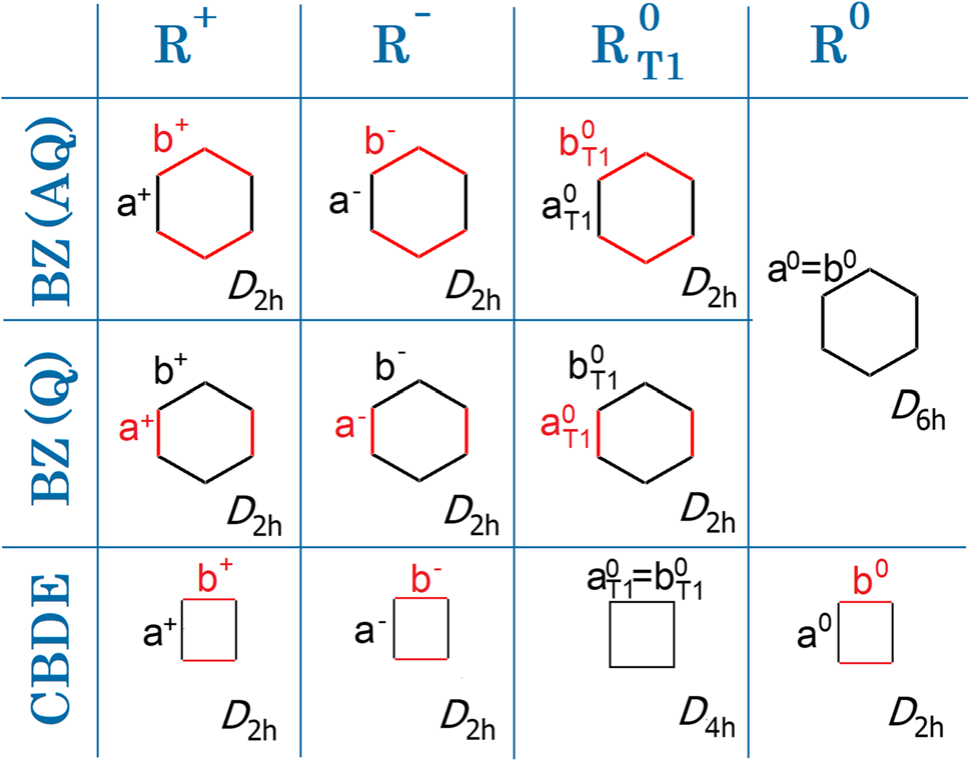
Molecular symmetry and C–C bond length labeling for investigated electronic states in quinoid (Q) and anti-quinoid (AQ) isomers of benzene (BZ) species, as well as in cyclobutadiene (CBDE)

With the enormous advances of supercomputing power which took place during the last quarter of a century, it was tempting for us to check the validity of the GAH rule using higher-level computational methods. It should be realized that the calculations performed over two decades ago were done using only one DFT functional (B3LYP) and were lacking any systematic assessment of the quantitative and qualitative impact of the level of theory on the final result. Thus, it could be that the GAH rule does not hold at all, if calculations are performed on a sufficiently high level of ab initio theory! We have selected two molecules for this study, i.e., cyclobutadiene (CBDE) and benzene (BZ), as well as a number of levels of theory and basis sets. Why CBDE and BZ? First, these molecules stand for prototypical antiaromatic (4e) and aromatic (6e) systems, respectively. On the other hand, their first excited triplet states are aromatic (2e) and antiaromatic (4e), respectively. In other words, the perturbation associated with one electron singlet → triplet excitation flips the aromaticity entirely to its opposite, a true revolution in electronic properties. Secondly, both molecules are relatively small, which permits quantum mechanical calculations to be performed at a quite precise reference computational levels. Third, having a large HOMO/LUMO gap, these small systems seem to be ideally suited for such test, according to Ayers and Parr predictions [2]. Fourth, both systems are cyclic which introduces a certain constraint on their geometry due to persistent σ bond framework. And last but not the least, BZ in its two radical ion forms as well as in the excited triplet state offers *two* distinct minima to be independently studied; one corresponds to a quinoid type (with two short and four long C–C bonds) and another to anti-quinoid one (with four short and two long bonds), so each of those may be looked at separately (Fig. 1). Despite their small size, the two molecules selected for this study host multiple fascinating phenomena and constitute a playground for theoretical methods. Take benzene; with only 6 π orbitals, not just two Kekulé ones and three Dewar ones but as many as 175 well-defined covalent and ionic valence bond structures are possible [4]. This leads to a multiconfigurational character of the this molecule of immense complexity, despite its seemingly simple regular geometry. The delocalized π-electron component of benzene is stabilized by resonance, but is also destabilized by localizing distortions, what is unfortunately a much less acknowledged fact [5]. Ulusoy and Nest showed that the aromaticity of benzene in its electronic ground state can simply be switched off by an ultrashort laser pulse [6]. The antiaromatic triplet state of benzene also exhibits many unusual complexities [7–11]. Clearly, there is still a lot to be learned from these small molecules.

## Computational details

For CBDE, four species were studied: singlet ground state, the first excited triplet state, radical cation, and radical anion. For BZ, each of the latter three was computed in two quinoid (Q) and anti-quinoid (AQ) form, thus leading to a total of seven distinct species.^1^ In the spirit of the original paper [1], we are looking at perturbations involving only the π manifold, free from, any π–σ coupling.^2^ Therefore, we perform geometry optimizations while constraining the planarity of each system (more on that and related complexities in the “Results and discussion” section). As a reasonable compromise between computational cost and quality of results, coupled-cluster (CC) based methodology was initially utilized in this study. This computational approach was assessed as very effective and accurate for theoretical researches of small and medium-sized organic molecules [12–14]. Therefore, we decided to use the linear response approximate coupled-cluster of second order (CC2) [15, 16] with efficient resolution-of-the-identity (RI) approximation [17, 18] implemented in Turbomole 7.1 package [19, 20]. CC2 computations were performed using Dunning and co-workers’ correlation-consistent basis sets (aug-)cc-pVXZ (X = D, T, Q) [21, 22]. In the case of CC2 computations, auxiliary basis sets [23] were also employed.

For comparison, all investigated structures were optimized also at (U)B3LYP [24–27], (U)M06-2X [28], and (U)CAM-B3LYP [29] density functional theory (DFT) levels of theory with Gaussian 16 revision A.03 [30]. Dunning’s cc-pVXZ (X = D, T, Q) [21, 22] and Pople’s 6-31G(d,p) basis sets were utilized [31]. *UltraFine* integration grid for numerical integrations and *Tight* geometry optimization criteria were used.

Finally, two multireference wave function-based approaches were also utilized: the complete active space self-consistent field method (CASSCF) [32–34] and the one based on its wavefunction second-order perturbation theory (CASPT2) [35, 36]. Both types of calculations were carried with Molcas 8.0 [37]. An active space was built with all π electrons and with 6 π-type orbitals, i.e., (6e, 6o) active space for neutral structures, (7e, 6o) for anionic and (5e, 6o) for cationic ones of BZ. Analogous active spaces for CBDE-based species also corresponded to all π electrons but now with 4 π-type orbitals. Considering the IPEA-shift parameter [38, 39], which modifies the zeroth-order Hamiltonian in the CASPT2 method, geometry optimizations were performed here with either the present-day default value of this parameter (*ε* = 0.25 a.u., S-IPEA) [40] or without using it at all (*ε* = 0.00 a.u., 0-IPEA).

To test the character of obtained stationary points and to cross-check the validity of constraint on the planarity of all species, vibrational frequencies were calculated with the same computational approach as for geometry optimization (except for all CASPT2 approaches and some of CASSCF ones, where computational cost exceeded resources available to us).

## Results and discussion

To utilize all described in the “Computational details” section computational approaches, the symmetry was constrained to *D*_6h_ for singlet BZ, *D*_4h_ for triplet CBDE, and *D*_2h_ for all other species. Thus, in each molecule, there are at most two distinct C–C bond lengths labeled as a and b (Fig. 1).

Each of those may in principle take different values for the ground singlet state in a neutral molecule, its first excited triplet state, as well as doublet states of the radical anion and radical cation, and they are labeled here as a^0^ or b^0^, a^0^_T1_ or b^0^_T1_, a^–^ or b^–^, and a^+^ or b^+^, respectively. The calculated bond lengths are collected in Table 1 (only for the largest basis sets studied here for each given level of theory, usually cc-pVQZ) and in Tables S1–S14 in Supplementary Information (an extended set for all basis sets studied).

**Table 1:** C–C bonds lengths [Å] in quinoid (Q) and anti-quinoid (AQ) conformers of benzene (BZ) and in cyclobutadiene (CBDE)

	Computational approach	a^+^	a^−^	a^0^_T1_	a^0^	ΔGAH(a)	b^+^	b^−^	b^0^_T1_	b^0^	ΔGAH(b)
BZ(AQ)	*CASPT2(ε* = *0.25)*	*1.447*	*1.456*	*1.499*	*1.393*	***0.011***	*1.387*	*1.395*	*1.392*	*1.393*	** − *0.003***
	CASSCF	1.440	1.452	1.499	1.392	**0.001**	1.383	1.392	1.391	1.392	** − 0.008**
	CC2	1.441	1.443	1.503	1.394	** − 0.012**	1.370	1.395	1.390	1.394	** − 0.018**
	DFT(B3LYP)	1.447	1.456	1.517	1.391	** − 0.004**	1.383	1.393	1.382	1.391	**0.003**
	DFT(M06-2X)	1.446	1.452	1.514	1.388	**-0.003**	1.380	1.389	1.379	1.388	**0.002**
	DFT(CAM-B3LYP)	1.443	1.451	1.513	1.385	**-0.005**	1.377	1.386	1.377	1.385	**0.001**
	max(**R**) – min(**R**)	0.007	0.013	0.018	0.009	**–-**	0.017	0.009	0.015	0.009	**–-**
BZ(Q)	*CASPT2(ε* = *0.25)*	*1.370*	*1.378*	*1.358*	*1.393*	***-0.003***	*1.426*	*1.435*	*1.463*	*1.393*	***0.005***
	CASSCF	1.364	1.373	1.353	1.392	**-0.008**	1.421	1.431	1.466	1.392	**-0.004**
	CC2	1.356	1.380	1.355	1.394	**-0.013**	1.412	1.432	1.464	1.394	**-0.014**
	DFT(B3LYP)	1.364	1.374	1.341	1.391	**0.007**	1.425	1.434	1.470	1.391	**-0.002**
	DFT(M06-2X)	1.360	1.370	1.335	1.388	**0.007**	1.424	1.430	1.470	1.388	**-0.003**
	DFT(CAM-B3LYP)	1.357	1.367	1.333	1.385	**0.006**	1.420	1.428	1.467	1.385	**-0.003**
	max(**R**) – min(**R**)	0.014	0.013	0.025	0.009	**–-**	0.014	0.007	0.007	0.009	**–-**
CBDE	*CASPT2(ε* = *0.25)*	*1.500*	*1.512*	*1.438*	*1.557*	***0.017***	*1.380*	*1.398*	*1.438*	*1.350*	***-0.010***
	CASSCF	1.482	1.499	1.435	1.547	**-0.001**	1.374	1.390	1.435	1.346	**-0.017**
	CC2	1.492	1.505	1.435	1.561	**0.002**	1.378	1.395	1.435	1.340	**-0.001**
	DFT(B3LYP)	1.500	1.512	1.436	1.574	**0.002**	1.373	1.390	1.436	1.329	**-0.002**
	DFT(M06-2X)	1.494	1.505	1.430	1.566	**0.003**	1.369	1.385	1.430	1.325	**-0.001**
	DFT(CAM-B3LYP)	1.493	1.504	1.429	1.566	**0.003**	1.367	1.384	1.429	1.323	**-0.001**
	max(**R**) – min(**R**)	0.018	0.013	0.009	0.027	**–-**	0.013	0.014	0.009	0.027	**–-**

For bond labeling for molecules in investigated electronic states see Fig. 1. ΔGAH(*R*) values [Å] (**bold**) were calculated according to Eq. 1. Computational data for the most resource-demanding CASPT2 approach are for cc-pVTZ basis set (*italics*), whereas all others are for cc-pVQZ. All values are rounded to three decimal places. Results obtained for other investigated basis sets are available in ESI. max(*R*)—min(R), where *R* = a or b, denotes the span of the bond length values between all methods tested for each molecular species and each bond separately

We begin by noticing that the predicted C–C bond lengths for each species separately are quite dependent mainly on the computational method (*cf.* SI). While, the C–C bond length in singlet BZ, a^0^, varies between 1.385 Å and 1.409 Å (i.e., by 0.024 Å), the discrepancy for its triplet state is much larger (0.049 Å for bond length a^0^_T1_). For CBDE, the smallest and the largest discrepancies are observed for radical anion (0.025 Å for bond length a^–^) and neutral singlet form (0.078 Å for b^0^), respectively.

Obviously, in our analysis, we will seek validation of Eq. 1 for one given level of theory at a time.

It is quite disturbing that the diverse computational methods often disagree on whether a planar geometry studied here is a local minimum or not. For high symmetry systems with “simple” electronic structure (triplet and singlet CBDE and singlet BZ), we find nearly no discrepancies, as expected. Still, the ground state benzene singlet turns out to show one imaginary frequency at CC2/aug-cc-pVTZ level and quite appreciable one (i423 cm^–1^) (*sic!*). The computational methods consistently yield the local minimum nature of the Q form of the BZ radical cation and for the CBDE radical cation. Yet, for the AQ form of BZ radical cation, we find that all DFT(M06-2X) calculations predict a transition state nature for this planar species, in contrast to all remaining methods.^3^^,^ 4 But the remaining open-shell species yield a true diversity of the number of imaginary frequencies. For example, the problematic BZ radical anion in its Q form is either a local minimum (according to selected DFT approaches), a transition state (selected CASSCF and DFT results), or a saddle point of the second order (according to the remaining CASSCF and DFT approaches). Similar problems are also encountered for the BZ triplet state (mostly in the Q form). What is even more puzzling, though, is that the CBDE radical anion (which, as a 3-electron 4-center system *must* host the Jahn–Teller effect in *D*_*4h*_ geometry) is computed by some DFT methods (e.g., CAM-B3LYP with either 6-31G(d,p) or cc-pVDZ basis set) to be an undistorted square. These problems are not unprecedented. The sensitivity of the potential energy surface details to the level of theory for these species is fascinating [41] and certainly comes from the fact that for relatively small 4- and 6-electron systems any 1e perturbation leading to a free radical may be considered large. The discrepancies between various methods document the problems which a theoretician faces while trying to correctly predict the geometry of a ground state. Fortunately, our task to verify the validity of the GAH rule for the pure π manifold (i.e. free from admixture of σ one) permits us to comfortably neglect these issues by restricting ourselves to planar geometries, independent of their character (i.e. a true minimum or not). Moreover, as wave-function-based methods are usually more sensitive than DFT ones to the choice of a basis set, from now on we discuss hereonly results obtained with the largest basis set (cc-pVQZ, except for the most resource-demanding CASPT2 where cc-pVTZ was used). For the remaining ones *cf.* the SI.

## Cyclobutadiene—antiaromatic system

**Fig. 2:**
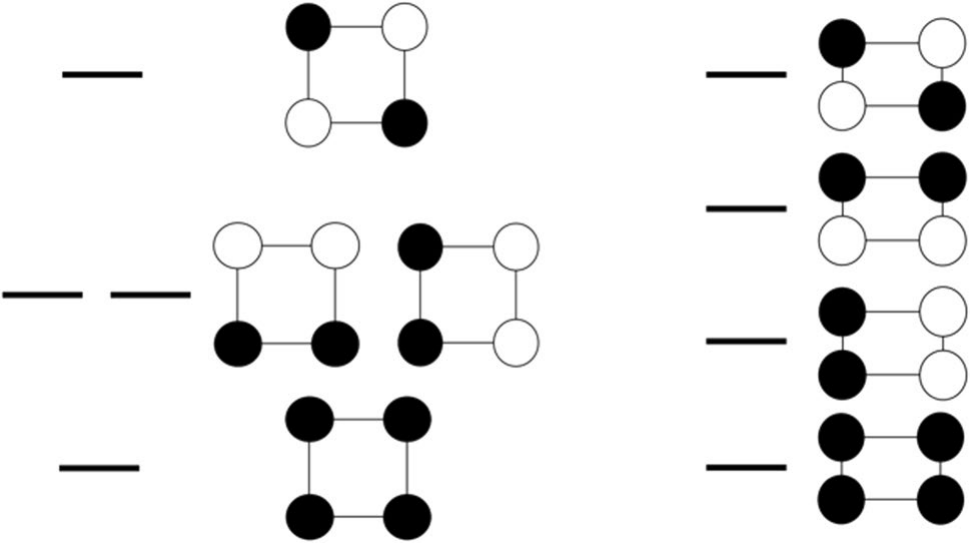
Illustration of the MO system of CBDE in *D*_*4h*_ geometry (left) and in a lower *D*_*2h*_ one (right). The rectangular deformation of a square has been exaggerated to facilitate the detection of bond length changes

Let us begin with the smaller and less complex of two systems, i.e. CBDE. The molecular orbital (MO) system of the π manifold for an ideal square *D*_*4h*_ symmetry is shown in Fig. 2 (left). The lowest energy orbital is nondegenerate, and it is bonding between all pairs of C atoms. Its fully antibonding equivalent is also nondegenerate, and obviously, it has the smallest binding energy. On the other hand, there are two different but energy-equivalent combinations of atomic orbitals, which are bonding between two pairs of C atoms and antibonding between the remaining two pairs. The triplet state of CBDE corresponds to a single occupation of each of the two degenerate orbitals and as such preserves *D*_*4h*_ symmetry. However, an occupation of these two orbitals by either one electron (in a radical cation), three electrons (in a radical anion), or one electron pair (in a singlet state) corresponds to the Jahn–Teller scenario, and it must lead to deformation of a square to a rectangle (*D*_*2h*_). This removes the orbital degeneracy (Fig. 2 right). Thus, a singlet ground state of CBDE is a prototypical antiaromatic system which exhibits bond alternation. This behavior may be formulated in terms of the maximum hardness principle [42–45]; the singlet state with one doubly filled and one empty degenerate orbitals would exhibit a null electronic gap at the Fermi level and infinite polarizability. The rectangular distortion leads to an increase of electronic hardness via band gap opening.

Since the changes of molecular geometry (i.e. short-long C–C bond pattern) are interconnected with the bonding/antibonding character of the two frontier orbitals, one may expect the following inequalities to hold:

2 $${\mathrm{a}}^{0}>{\mathrm{a}}^{-}\approx {\mathrm{a}}^{+}>{\mathrm{a}}_{\mathrm{T}1}^{0}$$

for a bond of CBDE

3 $${\mathrm{b}}^{0}<{\mathrm{b}}^{-}\approx {\mathrm{b}}^{+}<{\mathrm{b}}_{\mathrm{T}1}^{0}$$

for b bond of CBDE

This is because double occupancy of an orbital which is antibonding between a given pair of C atoms leads to a larger lengthening of the bond than a single occupation of the same orbital, and this in turn leads to a larger lengthening of the bond than for the unoccupied case.

Indeed, we notice that according to CASPT2, CASSCF, and CC2, the expected following inequalities consistently hold. However, all density-based approaches fail to show the expected bond length pattern for bond length b.

**Fig. 3:**
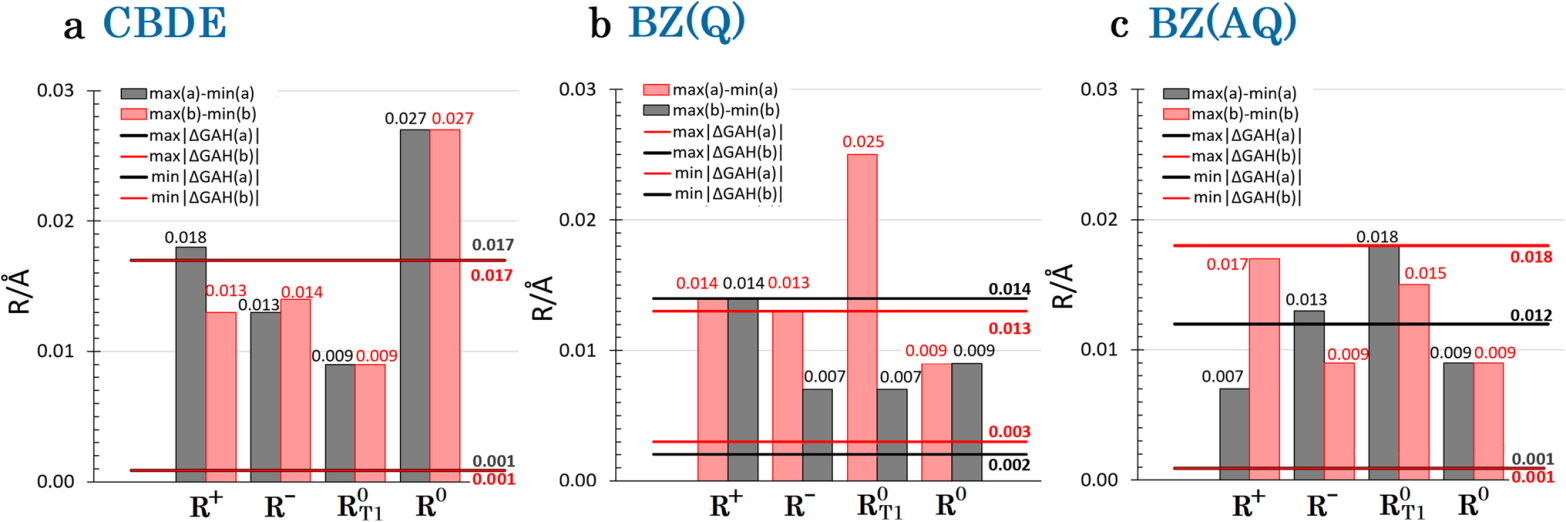
Illustration of the MO system of BZ in *D*_*6h*_ geometry; in Q and AQ structures of the radical cation, radical anion, and of the triplet state, the symmetry is lowered to *D*_*2h*_ (*cf.* Figure 1)

How well does the GAH rule hold for CBDE? It turns out that all methods wavefunction-based methods, ΔGAH(R) (Eq. 1) does not exceed + / − 0.017 Å (Fig. 3). This is not a lot, given that these methods show discrepancies for individual bonds of a similar magnitude, i.e. up to 0.018 Å (Table 1). In one particular case, that of CC2 calculations, the ΔGAH(a) is as little as 0.002 Å, while ΔGAH(b) equals − 0.001 Å. This implies that a “hybrid species” constructed with the help of the GAH rule from the ground singlet as well as radical anion and radical cation, not only closely resembles an undistorted square, but also the CC-bond lengths of this hybrid fall extremely close to that calculated for the triplet state (*D*_*4h*_).

**Fig. 4:**
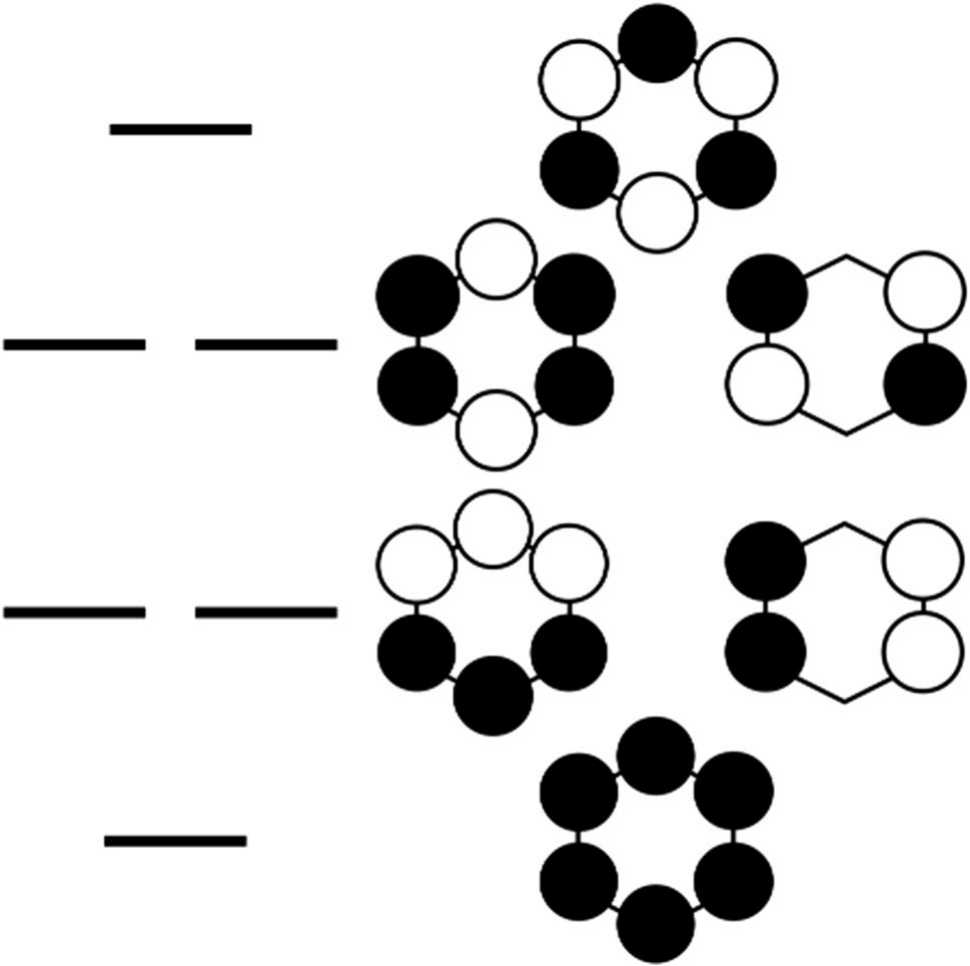
Minimum (min|ΔGAH(R)|) and maximum (max|ΔGAH(R)|) of absolute values of Eq. 1 expression max|ΔGAH(R)| on the background of statistic ranges max(R)-min(R) of a and b bonds lengths of quinoid (Q) and anti-quinoid (AQ) benzene (BZ) conformers and for cyclobutadiene (CBDE) for all investigated computational approaches and only with the use of the largest used basis sets (cc-pVTZ for CASPT2 computations and cc-pVQZ for other approaches, for numerical data, see Table 1). For statistics including results obtained with all basis sets see Fig. S1. Data for shorter bond are marked as red of pink, whereas data for longer ones are black or grey (see Fig. 1)

## Benzene—aromatic system

Let us now turn to a prototypical aromatic six-electron system—benzene. The MO scheme for BZ is illustrated in Fig. 4. The π manifold for an ideal hexagonal *D*_*6h*_ symmetry includes a totally-bonding nondegenerate orbital of the lowest energy, its nondegenerate totally-antibonding analog of the highest energy, and two pairs of degenerate MO sets. For 6e occupancy in the ground singlet state, the molecule shows an appreciable band gap among the two sets of the frontier orbitals; it is not subject to the Jahn–Teller effect, and it does not lower its symmetry. However, for either radical cation, radical anion or the lowest triplet state, the Jahn–Teller effect is operative, and the molecule distorts to *D*_*2h*_. There are two local energy minima depending on the phase of the Jahn–Teller distortion: a quinoid one (Q) with two short and four longer CC bonds and the antiquinoid one (AQ) with four short and two longer bonds. In such a case, the degeneracy of each previously doubly degenerate MO set is lifted. Obviously, this is also expected based on the maximum hardness principle.

**Table 2:** Mulliken atomic spin densities for carbon atoms of investigated ring molecules obtained at chosen computational approaches

	Computational approach	C_ab_(R^+^)	C_ab_(R^−^)	C_ab_(R^0^_T1_)	C_ab_(R^0^)	C_bb_(R^+^)	C_bb_(R^−^)	C_bb_(R^0^_T1_)	C_bb_(R^0^)
BZ(AQ)	*CASPT2(ε* = *0.25)*	*0.2894*	*0.2812*	*0.5341*	***0.0365***	* − 0.0844*	* − 0.0735*	* − 0.0830*	*** − 0.0749***
	CASSCF	0.2868	0.2769	0.5316	**0.0321**	− 0.0803	− 0.0738	− 0.0826	** − 0.0715**
	DFT(B3LYP)	0.2387	0.2331	0.6258	** − 0.1540**	0.0136	0.0067	− 0.1972	**0.2175**
	DFT(M06-2X)	0.2389	0.2326	0.6017	** − 0.1302**	0.0155	0.0143	− 0.2264	**0.2562**
	DFT(CAM-B3LYP)	0.2389	0.2325	0.6487	** − 0.1773**	0.0140	0.0100	− 0.2387	**0.2627**
BZ(Q)	*CASPT2(ε* = *0.25)*	0.0396	0.0407	0.1106	** − 0.0303**	0.4155	0.4073	0.7638	**0.0590**
	CASSCF	0.0414	0.0383	0.1053	** − 0.0256**	0.4106	0.4029	0.7697	**0.0438**
	DFT(B3LYP)	0.0740	0.0681	0.1017	**0.0404**	0.3430	0.3368	0.8508	** − 0.1710**
	DFT(M06-2X)	0.0766	0.0722	0.0800	**0.0687**	0.3403	0.3352	0.8071	** − 0.1317**
	DFT(CAM-B3LYP)	0.0745	0.0694	0.0884	**0.0555**	0.3430	0.3361	0.8814	** − 0.2024**
CBDE	*CASPT2(ε* = *0.25)*	0.2472	0.2451	0.4927	** − 0.0004**				
	CASSCF	0.2493	0.2489	0.4981	**0.0001**				
	DFT(B3LYP)	0.2484	0.2478	0.4961	**0.0001**				
	DFT(M06-2X)	0.2471	0.2450	0.4925	** − 0.0004**				
	DFT(CAM-B3LYP)	0.2466	0.2404	0.4896	** − 0.0026**				

Because of molecular symmetry, we present here values for two unique carbon atoms in BZ (forming a and b bonds—marked as C_ab_ and forming two b bonds—marked as C_bb_, see Fig. 1) and for one in CBDE (C_ab_). Values for ground electronic state C(R^0^) (**bold**) were predicted as a combination C(R^+^) + C(R^−^) − C(R^0^_T1_) of atomic spin densities computed for other electronic state structures. Please note that data for the CASPT2 method are computed for equilibrium geometries obtained at this level of theory; however, spin densities are computed basing on CASSCF wavefunction. All values are rounded to three decimal places and where necessary averaged because of molecular symmetry. Results obtained for other investigated basis sets are available in ESI

**Table 3:** Mulliken atomic spin densities for carbon atoms (atomic spin densities of hydrogens are summed into C atoms they are connected to) of investigated ring molecules obtained at chosen computational approaches

	Computational approach	C_ab_(R^+^)	C_ab_(R^−^)	C_ab_(R^0^_T1_)	C_ab_(R^0^)	C_bb_(R^+^)	C_bb_(R^−^)	C_bb_(R^0^_T1_)	C_bb_(R^0^)
BZ(AQ)	*CASPT2(ε* = *0.25)*	*0.2925*	*0.2871*	*0.5419*	***0.0377***	* − 0.0852*	* − 0.0743*	* − 0.0838*	*** − 0.0757***
	CASSCF	0.2906	0.2873	0.5418	**0.0361**	− 0.0813	− 0.0745	− 0.0835	** − 0.0723**
	DFT(B3LYP)	0.2432	0.2465	0.5970	** − 0.1073**	0.0136	0.0069	− 0.1940	**0.2145**
	DFT(M06-2X)	0.2423	0.2428	0.6053	** − 0.1203**	0.0155	0.0145	− 0.2107	**0.2406**
	DFT(CAM-B3LYP)	0.2430	0.2449	0.6159	** − 0.1280**	0.0155	0.0102	− 0.2317	**0.2559**
BZ(Q)	*CASPT2(ε* = *0.25)*	0.0400	0.0419	0.1124	** − 0.0305**	0.4199	0.4162	0.7752	**0.0609**
	CASSCF	0.0420	0.0407	0.1076	** − 0.0249**	0.4161	0.4186	0.7847	**0.0500**
	DFT(B3LYP)	0.0753	0.0722	0.0926	**0.0549**	0.3494	0.3556	0.8148	** − 0.1098**
	DFT(M06-2X)	0.0776	0.0754	0.0927	**0.0602**	0.3449	0.3493	0.8145	** − 0.1204**
	DFT(CAM-B3LYP)	0.0756	0.0732	0.0804	**0.0684**	0.3488	0.3535	0.8391	** − 0.1369**
CBDE	*CASPT2(ε* = *0.25)*	0.2500	0.2500	0.5000	**0.0000**				
	CASSCF	0.2500	0.2500	0.5000	**0.0000**				
	DFT(B3LYP)	0.2500	0.2500	0.5000	**0.0000**				
	DFT(M06-2X)	0.2500	0.2500	0.5000	**0.0000**				
	DFT(CAM-B3LYP)	0.2500	0.2500	0.5000	**0.0000**				

Because of molecular symmetry, we present here values for two unique carbon atoms in BZ (forming a and b bonds—marked as C_ab_ and forming two b bonds—marked as C_bb_, see Fig. 1) and for one in CBDE (C_ab_). Values for ground electronic state C(R^0^) (**bold**) were predicted as a combination of C(R^+^) + C(R) − C(R^0^_T1_) of atomic spin densities computed for other electronic state structures. Please note that data for the CASPT2 method are computed for equilibrium geometries obtained at this level of theory; however, spin densities are computed basing on CASSCF wavefunction. All values are rounded to three decimal places and where necessary averaged because of molecular symmetry. Results obtained for other investigated basis sets are available in ESI

## Conclusions

**Fig. 5:**
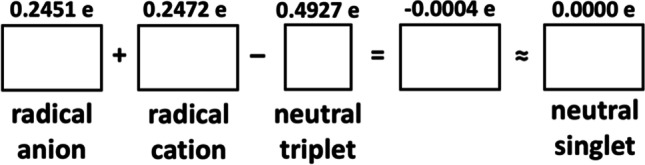
Illustration of the applicability of the GAH rule to atomic spin densities on C atoms for CBDE at the CASPT2 level (cf. Table 2). The rectangular deformation of a square has been exaggerated

We have reinvestigated the applicability of a simplistic bond length rule (GAH rule) for two cyclic molecules, CBDE and BZ, as prototypical examples of anti-aromatic and aromatic molecules, respectively, and using modern computational approaches of quantum chemistry. The rule was tested in its original spirit, i.e., assuming an enforced planar geometry of the carbon rings for all species considered. In general, the GAH rule is reasonably satisfied for these molecules, with its errors not surpassing 0.018 Å for wavefunction-based methods and large basis sets (Fig. 3). Moreover, the rule is reasonably fulfilled for atomic spin densities (Table 2 and 3), particularly at CASSCF and CASPT2 levels, leading to rather small residual spin densities (typically + / − 0.01 e and up to + / − 0.07 e in some cases) for hybrids of radical anion, radical cation and triplet state, thus resembling a spin-less singlet state (Fig. 5).

It is interesting that the rule seems to hold particularly due to the fact that the radical anion species is frozen here in the planar geometry, while it usually tries to break planarity for very small systems such as BZ or CBDE [12]. On the other hand, it is known that much larger aromatic hydrocarbons (or smaller, but fluorinated ones [46]) show positive electron affinity, and they tend to form stable and planar radical anions; hence, one might anticipate that the rule will apply very well for such systems. Yet, computational verification of the applicability of the rule for large systems requires more approximate quantum chemistry tools than those applied here, and this will be investigated in the near future. It seems that the growing interest in the structure and properties of excited states of small organic molecules [47] warrants further studies in this direction.

## Supplementary information

Below is the link to the electronic supplementary material.

Supplementary file1 (PDF 2220 KB)

## Data Availability

Data obtained from computations described in the current study are available from the corresponding author upon reasonable request.

## Footnotes

1 Here, we neglect the fact that BZ radical cation is a very flexible system, which at most experimental conditions is able to tunel through low barriers between these minima thus forming an apparently higher-symmetry *D*_6h_ structure.

2 Importance of such coupling may vary greatly from one species to the other; for example, radical anion of benzene, coupled with K^+^(crown ether) cations, in the solid state, tends to loose planarity and subsequently couple two radical anions via a new C–C sigma bond.

3 Note that radical anion of BZ and CBDE are immensely difficult to be correctly described by comprehensive theory with a limited basis set. *cf.* [48]. Aside from deplanarization, these species tend to dimerize when prepared in experiment, see e.g.: [49]. Alternative approach to avoid dimerization involves use of protective groups: [50].

4 Interestingly, calculations find also a rhomboidal minimum for CBDE radical anion; rhomboidal distortion rather than bond length alternation is an alternative way to avoid the Jahn–Teller effect.
