# Supplementary material for: Quadripartite bond length rule applied to two prototypical aromatic and antiaromatic molecules
Source: J Mol Model. 2023 Mar 13;29(4):95. doi: 10.1007/s00894-023-05498-4 (PMC10008775; doi:10.1007/s00894-023-05498-4)
Supplement: Supplementary file 1 — Supplementary file1 (PDF 2220 KB) [file 894_2023_5498_MOESM1_ESM.pdf]

SUPPLEMENTARY INFORMATION

Quadripartite bond length rule applied to two prototypical aromatic and antiaromatic molecules

Łukasz Wolański and Wojciech Grochala**

Centre of New Technologies, University of Warsaw, S. Banacha 2c, 02-097 Warsaw, Poland

* e-mail: l.wolanski@cent.uw.edu.pl, w.grochala@cent.uw.edu.pl

Table of contents

**Table S1.**	Selected geometrical parameters of anionic form of BZ(Q)	S3
**Table S2.**	Selected geometrical parameters of anionic form of BZ(AQ)	S4
**Table S3.**	Selected geometrical parameters of cationic form of BZ(Q)	S5
**Table S4.**	Selected geometrical parameters of cationic form of BZ(AQ)	S6
**Table S5.**	Selected geometrical parameters of ground state neutral form of BZ	S7
**Table S6.**	Selected geometrical parameters of first triplet excited state neutral form of BZ(Q)	S8
**Table S7.**	Selected geometrical parameters of first triplet excited state neutral form of BZ(AQ)	S9
**Table S8.**	ΔGAH(R) values for selected bonds of BZ(Q)	S10
**Table S9.**	ΔGAH(R) values for selected bonds of BZ(AQ)	S11
**Table S10.**	Selected geometrical parameters of anionic form of CBDE	S12
**Table S11.**	Selected geometrical parameters of cationic form of CBDE	S13
**Table S12.**	Selected geometrical parameters of ground state neutral form of CBDE	S14
**Table S13.**	Selected geometrical parameters of first triplet excited state neutral form of CBDE	S15
**Table S14.**	ΔGAH(R) values for selected bonds of CBDE	S16
**Table S15.**	Selected geometrical parameters of neutral radical form of CP	S17
**Table S16.**	Selected geometrical parameters of bicationic radical form of CP	S17
**Table S17.**	Selected geometrical parameters of ground state cationic form of CP	S18
**Table S18.**	Selected geometrical parameters of first triplet excited state cationic form of CP	S18
**Table S19.**	Mulliken atomic spin densities for anionic form of BZ(Q)	S19
**Table S20.**	Mulliken atomic spin densities for anionic form of BZ(AQ)	S20
**Table S21.**	Mulliken atomic spin densities for cationic form of BZ(Q)	S21
**Table S22.**	Mulliken atomic spin densities for cationic form of BZ(AQ)	S22
**Table S23.**	Mulliken atomic spin densities for first triplet excited state neutral form of BZ(Q)	S23
**Table S24.**	Mulliken atomic spin densities for first triplet excited state neutral form of BZ(AQ)	S24
**Table S25.**	GAH-rule based atomic spin densities for ground state neutral BZ (from Q-forms)	S25
**Table S26.**	GAH-rule based atomic spin densities for ground state neutral BZ (from AQ-forms)	S26
**Table S27.**	Mulliken atomic spin densities for anionic form of CBDE	S27
**Table S28.**	Mulliken atomic spin densities for cationic form of CBDE	S28
**Table S29.**	Mulliken atomic spin densities for first triplet excited state neutral form of CBDE	S29
**Table S30.**	GAH-rule based Mulliken atomic spin densities for ground state neutral CBDE	S30
**Figure S1.**	Maximum unsigned values of ΔGAH(R) values on the background of statistic ranges max(R)-min(R)	S31

Table S1. Selected bond lengths [Å] and valence bond angles [°] between carbon atoms in quinoid-like anionic variant of benzene ring obtained from chosen approaches of quantum chemistry computational methods. C-C bond are labelled by designations introduced by **Figure 1**. If it is indicated, vibrational analysis was carried out for the equilibrium structure. All obtained structures are flat, possible imaginary frequencies relate to the swing of hydrogen atoms off the plane of symmetry, or are numerical artefacts of DFT methodology.

Total charge: **-1**

Spin multiplicity: **2**

Geometry optimization state: **D_0**

BZ(Q) R^-

Method	Basis set	Bond lenghts [Å]		Valence bond angles [°]		Imag. freq.
		a^-	b^-	∠(a^-,b^-)	∠(b^-,b^-)	
CASPT2 (7e,6o), 0-IPEA	6-31G(d,p)	1.381	1.438	121.59	116.81	n/d
CASPT2 (7e,6o), 0-IPEA	cc-pVDZ	1.391	1.448	121.56	116.88	n/d
CASPT2 (7e,6o), 0-IPEA	cc-pVTZ	1.380	1.436	121.52	116.96	n/d
CASPT2 (7e,6o), S-IPEA	6-31G(d,p)	1.380	1.437	121.62	116.77	n/d
CASPT2 (7e,6o), S-IPEA	cc-pVDZ	1.390	1.447	121.59	116.83	n/d
CASPT2 (7e,6o), S-IPEA	cc-pVTZ	1.378	1.435	121.55	116.91	n/d
CASSCF (7e,6o)	6-31G(d,p)	1.376	1.436	121.64	116.73	2 [-286, -132 cm^{-1}]
CASSCF (7e,6o)	cc-pVDZ	1.379	1.438	121.60	116.79	1 [211 cm^{-1}]
CASSCF (7e,6o)	cc-pVTZ	1.373	1.432	121.57	116.86	1 [218 cm^{-1}]
CASSCF (7e,6o)	cc-pVQZ	1.373	1.431	121.54	116.92	2 [-264, -123 cm^{-1}]
CC2	cc-pVDZ	1.393	1.446	121.60	116.80	2 [-294, -243 cm^{-1}]
CC2	aug-cc-pVDZ	1.398	1.445	121.42	117.17	n/d
CC2	cc-pVTZ	1.381	1.435	121.55	116.90	2 [-276, -191 cm^{-1}]
CC2	aug-cc-pVTZ	1.385	1.431	121.39	117.22	n/d
CC2	cc-pVQZ	1.380	1.432	121.51	116.98	2 [-305, -238 cm^{-1}]
CC2	aug-cc-pVQZ	1.383	1.428	121.35	117.31	n/d
DFT(B3LYP)	6-31G(d,p)	1.378	1.440	121.53	116.94	1 [-252 cm^{-1}]
DFT(B3LYP)	cc-pVDZ	1.381	1.442	121.49	117.02	0
DFT(B3LYP)	cc-pVTZ	1.373	1.434	121.45	117.09	1 [-139 cm^{-1}]
DFT(B3LYP)	cc-pVQZ	1.374	1.434	121.43	117.13	2 [-231, -53 cm^{-1}]
DFT(M06-2X)	6-31G(d,p)	1.373	1.435	121.58	116.83	1 [-119 cm^{-1}]
DFT(M06-2X)	cc-pVDZ	1.377	1.437	121.54	116.92	0
DFT(M06-2X)	cc-pVTZ	1.370	1.431	121.51	116.98	0
DFT(M06-2X)	cc-pVQZ	1.370	1.430	121.48	117.04	1 [-54 cm^{-1}]
DFT(CAM-B3LYP)	6-31G(d,p)	1.371	1.434	121.55	116.90	1 [-125 cm^{-1}]
DFT(CAM-B3LYP)	cc-pVDZ	1.374	1.437	121.56	116.88	1 [-94 cm^{-1}]
DFT(CAM-B3LYP)	cc-pVTZ	1.366	1.429	121.47	117.05	0
DFT(CAM-B3LYP)	cc-pVQZ	1.367	1.428	121.45	117.09	1 [-131 cm^{-1}]

n/d - *not determined (because of computational cost or some numerical troubles)*

Table S2. Selected bond lengths [Å] and valence bond angles [°] between carbon atoms in anti-quinoid-like anionic variant of benzene ring obtained from chosen approaches of quantum chemistry computational methods. C-C bond are labelled by designations introduced by **Figure 1**. If it is indicated, vibrational analysis was carried out for the equilibrium structure. All obtained structures are flat, possible imaginary frequencies relate to the swing of hydrogen atoms off the plane of symmetry, or are numerical artefacts of DFT methodology.

Total charge: -1
Spin multiplicity: 2
Geometry optimization state: D_0

BZ(AQ) R^-

Method	Basis set	Bond lenghts [Å]		Valence bond angles [°]		Imag. freq.
		a^-	b^-	∠(a^-,b^-)	∠(b^-,b^-)	
CASPT2 (7e,6o), 0-IPEA	6-31G(d,p)	1.460	1.399	118.44	123.13	n/d
CASPT2 (7e,6o), 0-IPEA	cc-pVDZ	1.470	1.409	118.45	123.10	n/d
CASPT2 (7e,6o), 0-IPEA	cc-pVTZ	1.457	1.396	116.84	126.31	n/d
CASPT2 (7e,6o), S-IPEA	6-31G(d,p)	1.459	1.398	118.43	123.15	n/d
CASPT2 (7e,6o), S-IPEA	cc-pVDZ	1.468	1.407	118.44	123.12	n/d
CASPT2 (7e,6o), S-IPEA	cc-pVTZ	1.456	1.395	116.81	126.38	n/d
CASSCF (7e,6o)	6-31G(d,p)	1.457	1.394	118.45	123.09	1 [-182 cm^{-1}]
CASSCF (7e,6o)	cc-pVDZ	1.459	1.399	118.49	123.03	1 [-155 cm^{-1}]
CASSCF (7e,6o)	cc-pVTZ	1.453	1.392	118.52	122.96	1 [-188 cm^{-1}]
CASSCF (7e,6o)	cc-pVQZ	1.452	1.392	118.55	122.90	1 [-254 cm^{-1}]
CC2	cc-pVDZ	1.466	1.408	118.46	123.07	1 [-535 cm^{-1}]
CC2	aug-cc-pVDZ	1.461	1.412	118.68	122.65	n/d
CC2	cc-pVTZ	1.454	1.397	118.50	123.00	1 [-558 cm^{-1}]
CC2	aug-cc-pVTZ	1.447	1.399	118.70	122.60	n/d
CC2	cc-pVQZ	1.451	1.395	118.54	122.92	1 [-563 cm^{-1}]
CC2	aug-cc-pVQZ	1.443	1.396	118.75	122.51	n/d
DFT(B3LYP)	6-31G(d,p)	1.463	1.397	118.47	123.06	1 [-220 cm^{-1}]
DFT(B3LYP)	cc-pVDZ	1.465	1.400	118.51	122.98	1 [-215 cm^{-1}]
DFT(B3LYP)	cc-pVTZ	1.457	1.392	118.54	122.91	1 [-168 cm^{-1}]
DFT(B3LYP)	cc-pVQZ	1.456	1.393	118.57	122.56	1 [-168 cm^{-1}]
DFT(M06-2X)	6-31G(d,p)	1.458	1.392	118.41	123.18	1 [-322 cm^{-1}]
DFT(M06-2X)	cc-pVDZ	1.459	1.395	118.45	123.09	1 [-316 cm^{-1}]
DFT(M06-2X)	cc-pVTZ	1.454	1.389	118.49	123.01	1 [-295 cm^{-1}]
DFT(M06-2X)	cc-pVQZ	1.452	1.389	118.53	122.94	1 [-355 cm^{-1}]
DFT(CAM-B3LYP)	6-31G(d,p)	1.457	1.391	118.46	123.08	1 [-340 cm^{-1}]
DFT(CAM-B3LYP)	cc-pVDZ	1.460	1.394	118.48	123.05	0
DFT(CAM-B3LYP)	cc-pVTZ	1.452	1.386	118.54	122.93	1 [-314 cm^{-1}]
DFT(CAM-B3LYP)	cc-pVQZ	1.451	1.386	118.56	122.88	1 [-322 cm^{-1}]

n/d - *not determined (because of computational cost or some numerical troubles)*

Table S3. Selected bond lengths [Å] and valence bond angles [°] between carbon atoms in quinoid-like cationic variant of benzene ring obtained from chosen approaches of quantum chemistry computational methods. C-C bond are labelled by designations introduced by **Figure 1**. If it is indicated, vibrational analysis was carried out for the equilibrium structure. All obtained structures are flat, possible imaginary frequencies relate to the swing of hydrogen atoms off the plane of symmetry, or are numerical artefacts of DFT methodology.

Total charge: 1
Spin multiplicity: 2
Geometry optimization state: D_0

BZ(Q) R⁺

Method	Basis set	Bond lenghts [Å]		Valence bond angles [°]		Imag. freq.
		a^+	b^+	∠(a^+,b^+)	∠(b^+,b^+)	
CASPT2 (5e,6o), 0-IPEA	6-31G(d,p)	1.376	1.430	119.16	121.66	n/d
CASPT2 (5e,6o), 0-IPEA	cc-pVDZ	1.385	1.438	119.15	121.71	n/d
CASPT2 (5e,6o), 0-IPEA	cc-pVTZ	1.372	1.426	119.13	121.74	n/d
CASPT2 (5e,6o), S-IPEA	6-31G(d,p)	1.375	1.429	119.16	121.68	n/d
CASPT2 (5e,6o), S-IPEA	cc-pVDZ	1.384	1.438	119.14	121.72	n/d
CASPT2 (5e,6o), S-IPEA	cc-pVTZ	1.370	1.426	119.12	121.75	n/d
CASSCF (5e,6o)	6-31G(d,p)	1.370	1.426	119.26	121.49	0
CASSCF (5e,6o)	cc-pVDZ	1.372	1.427	119.24	121.52	0
CASSCF (5e,6o)	cc-pVTZ	1.365	1.422	119.24	121.52	0
CASSCF (5e,6o)	cc-pVQZ	1.364	1.421	119.24	121.52	0
CC2	cc-pVDZ	1.373	1.426	124.06	111.99	0
CC2	aug-cc-pVDZ	1.373	1.428	124.08	111.85	0
CC2	cc-pVTZ	1.359	1.414	124.08	111.83	0
CC2	aug-cc-pVTZ	1.359	1.415	124.08	111.84	0
CC2	cc-pVQZ	1.356	1.412	124.08	111.83	0
CC2	aug-cc-pVQZ	1.357	1.413	124.07	111.86	0
DFT(B3LYP)	6-31G(d,p)	1.372	1.432	119.18	121.65	0
DFT(B3LYP)	cc-pVDZ	1.374	1.433	119.17	121.65	0
DFT(B3LYP)	cc-pVTZ	1.365	1.426	119.18	121.64	0
DFT(B3LYP)	cc-pVQZ	1.364	1.426	119.18	121.64	0
DFT(M06-2X)	6-31G(d,p)	1.367	1.430	119.12	121.77	0
DFT(M06-2X)	cc-pVDZ	1.368	1.431	119.10	121.80	0
DFT(M06-2X)	cc-pVTZ	1.360	1.425	119.10	121.80	0
DFT(M06-2X)	cc-pVQZ	1.360	1.424	119.11	121.78	0
DFT(CAM-B3LYP)	6-31G(d,p)	1.365	1.427	119.12	121.77	0
DFT(CAM-B3LYP)	cc-pVDZ	1.367	1.428	119.11	121.78	0
DFT(CAM-B3LYP)	cc-pVTZ	1.358	1.421	119.12	121.77	0
DFT(CAM-B3LYP)	cc-pVQZ	1.357	1.420	119.12	121.77	0

n/d - *not determined (because of computational cost or some numerical troubles)*

Table S4. Selected bond lengths [Å] and valence bond angles [°] between carbon atoms in antiquinoid-like cationic variant of benzene ring obtained from chosen approaches of quantum chemistry computational methods. C-C bond are labelled by designations introduced by **Figure 1**. If it is indicated, vibrational analysis was carried out for the equilibrium structure. All obtained structures are flat, possible imaginary frequencies relate to the swing of hydrogen atoms off the plane of symmetry, or are numerical artefacts of DFT methodology.

Total charge: 1

Spin multiplicity: 2

Geometry optimization state: D_0

BZ(AQ) R^+

Method	Basis set	Bond lenghts [Å]		Valence bond angles [°]		Imag. freq.
		a^+	b^+	∠(a^+,b^+)	∠(b^+,b^+)	
CASPT2 (5e,6o), 0-IPEA	6-31G(d,p)	1.451	1.393	120.83	118.34	n/d
CASPT2 (5e,6o), 0-IPEA	cc-pVDZ	1.459	1.402	120.84	118.31	n/d
CASPT2 (5e,6o), 0-IPEA	cc-pVTZ	1.448	1.389	120.86	118.28	n/d
CASPT2 (5e,6o), S-IPEA	6-31G(d,p)	1.450	1.392	120.84	118.31	n/d
CASPT2 (5e,6o), S-IPEA	cc-pVDZ	1.458	1.400	120.86	118.26	n/d
CASPT2 (5e,6o), S-IPEA	cc-pVTZ	1.447	1.387	120.87	118.25	n/d
CASSCF (5e,6o)	6-31G(d,p)	1.444	1.389	120.81	118.38	1 [-436 cm^{-1}]
CASSCF (5e,6o)	cc-pVDZ	1.445	1.390	120.81	118.35	1 [-417 cm^{-1}]
CASSCF (5e,6o)	cc-pVTZ	1.440	1.384	120.83	118.35	1 [-378 cm^{-1}]
CASSCF (5e,6o)	cc-pVQZ	1.440	1.383	120.83	118.35	1 [-406 cm-1]
CC2	cc-pVDZ	1.455	1.386	116.14	127.72	1 [-1049 cm^{-1}]
CC2	aug-cc-pVDZ	1.457	1.387	116.09	127.83	1 [-1032 cm^{-1}]
CC2	cc-pVTZ	1.444	1.373	116.07	127.87	1 [-1083 cm^{-1}]
CC2	aug-cc-pVTZ	1.444	1.373	116.05	127.91	2 [-1074, -522 cm^{-1}]
CC2	cc-pVQZ	1.441	1.370	116.06	127.87	1 [-1092 cm^{-1}]
CC2	aug-cc-pVQZ	1.441	1.371	116.07	127.86	n/d
DFT(B3LYP)	6-31G(d,p)	1.453	1.391	120.82	118.35	1 [-436 cm^{-1}]
DFT(B3LYP)	cc-pVDZ	1.455	1.392	120.82	118.36	1 [-434 cm^{-1}]
DFT(B3LYP)	cc-pVTZ	1.448	1.384	120.81	118.37	1 [-410 cm^{-1}]
DFT(B3LYP)	cc-pVQZ	1.447	1.383	120.81	118.37	1 [-409 cm-1]
DFT(M06-2X)	6-31G(d,p)	1.451	1.386	120.93	118.13	1 [-1213 cm^{-1}]
DFT(M06-2X)	cc-pVDZ	1.452	1.388	120.94	118.12	1 [-1166 cm^{-1}]
DFT(M06-2X)	cc-pVTZ	1.447	1.380	120.94	118.12	1 [-1087 cm^{-1}]
DFT(M06-2X)	cc-pVQZ	1.446	1.380	120.93	118.13	1 [-1114 cm^{-1}]
DFT(CAM-B3LYP)	6-31G(d,p)	1.448	1.384	120.90	118.20	1 [-395 cm^{-1}]
DFT(CAM-B3LYP)	cc-pVDZ	1.450	1.386	120.90	118.20	1 [-387 cm^{-1}]
DFT(CAM-B3LYP)	cc-pVTZ	1.443	1.377	120.89	118.21	1 [-370 cm^{-1}]
DFT(CAM-B3LYP)	cc-pVQZ	1.443	1.377	120.89	118.21	1 [-366 cm^{-1}]

n/d - *not determined (because of computational cost or some numerical troubles)*

Table S5. Selected bond lengths [Å] and valence bond angles [^{o}] between carbon atoms in neutral variant of benzene ring obtained from chosen approaches of quantum chemistry computational methods. C-C bond are labelled by designations introduced by **Figure 1**. If it is indicated, vibrational analysis was carried out for the equilibrium structure. All obtained structures are flat, possible imaginary frequencies relate to the swing of hydrogen atoms off the plane of symmetry, or are numerical artefacts of DFT methodology..

BZ R^0

Total charge: 0
Spin multiplicity: 1
Geometry optimization state: S_0

Method	Basis set	Bond lenght [Å] $a^0=b^0$	Valence bond angles [^{o}] $\angle(a^0=b^0, a^0=b^0)$	Imag. freq.
CASPT2 (6e,6o), 0-IPEA	6-31G(d,p)	1.398	120.00	n/d
CASPT2 (6e,6o), 0-IPEA	cc-pVDZ	1.407	120.00	n/d
CASPT2 (6e,6o), 0-IPEA	cc-pVTZ	1.395	120.00	n/d
CASPT2 (6e,6o), S-IPEA	6-31G(d,p)	1.397	120.00	n/d
CASPT2 (6e,6o), S-IPEA	cc-pVDZ	1.406	120.00	n/d
CASPT2 (6e,6o), S-IPEA	cc-pVTZ	1.393	120.00	n/d
CASSCF (6e,6o)	6-31G(d,p)	1.396	120.00	0
CASSCF (6e,6o)	cc-pVDZ	1.398	120.00	0
CASSCF (6e,6o)	cc-pVTZ	1.392	120.00	0
CASSCF (6e,6o)	cc-pVQZ	1.392	120.00	0
CC2	cc-pVDZ	1.407	120.00	0
CC2	aug-cc-pVDZ	1.409	120.00	0
CC2	cc-pVTZ	1.395	120.00	0
CC2	aug-cc-pVTZ	1.396	120.00	1 [-423 cm^{-1}]
CC2	cc-pVQZ	1.394	120.00	0
CC2	aug-cc-pVQZ	1.394	120.00	0
DFT(B3LYP)	6-31G(d,p)	1.396	120.00	0
DFT(B3LYP)	cc-pVDZ	1.399	120.00	0
DFT(B3LYP)	cc-pVTZ	1.391	120.00	0
DFT(B3LYP)	cc-pVQZ	1.391	120.00	0
DFT(M06-2X)	6-31G(d,p)	1.393	120.00	0
DFT(M06-2X)	cc-pVDZ	1.395	120.00	0
DFT(M06-2X)	cc-pVTZ	1.388	120.00	0
DFT(M06-2X)	cc-pVQZ	1.388	120.00	0
DFT(CAM-B3LYP)	6-31G(d,p)	1.391	120.00	0
DFT(CAM-B3LYP)	cc-pVDZ	1.393	120.00	0
DFT(CAM-B3LYP)	cc-pVTZ	1.385	120.00	0
DFT(CAM-B3LYP)	cc-pVQZ	1.385	120.00	0

n/d - *not determined (because of computational cost or some numerical troubles)*

Table S6. Selected bond lengths [Å] and valence bond angles [°] between carbon atoms in quinoid-like neutral variant of benzene ring in the first electronic triplet excided state obtained from chosen approaches of quantum chemistry computational methods. C-C bond are labelled by designations introduced by **Figure 1**. If it is indicated, vibrational analysis was carried out for the equilibrium structure. All obtained structures are flat, possible imaginary frequencies relate to the swing of hydrogen atoms off the plane of symmetry, or are numerical artefacts of DFT methodology.

Total charge: 0

Spin multiplicity: 1

Geometry optimization state: T_1

BZ(Q) R^0_{T1}

Method	Basis set	Bond lenghts [Å]		Valence bond angles [°]		Imag. freq.
		a^0_{T1}	b^0_{T1}	∠(a^0_{T1}, b^0_{T1})	∠(b^0_{T1}, b^0_{T1})	
CASPT2 (6e,6o), 0-IPEA	6-31G(d,p)	1.373	1.461	120.30	119.40	n/d
CASPT2 (6e,6o), 0-IPEA	cc-pVDZ	1.382	1.469	120.29	119.42	n/d
CASPT2 (6e,6o), 0-IPEA	cc-pVTZ	1.366	1.459	120.29	119.42	n/d
CASPT2 (6e,6o), S-IPEA	6-31G(d,p)	1.365	1.464	120.39	119.22	n/d
CASPT2 (6e,6o), S-IPEA	cc-pVDZ	1.373	1.473	120.38	119.23	n/d
CASPT2 (6e,6o), S-IPEA	cc-pVTZ	1.358	1.463	120.39	119.23	n/d
CASSCF (6e,6o)	6-31G(d,p)	1.361	1.468	120.52	118.97	n/d
CASSCF (6e,6o)	cc-pVDZ	1.363	1.470	120.51	118.98	n/d
CASSCF (6e,6o)	cc-pVTZ	1.354	1.466	120.53	118.94	n/d
CASSCF (6e,6o)	cc-pVQZ	1.353	1.466	120.53	118.93	n/d
CC2	cc-pVDZ	1.374	1.476	120.41	119.18	n/d
CC2	aug-cc-pVDZ	1.375	1.478	120.40	119.21	n/d
CC2	cc-pVTZ	1.358	1.466	120.42	119.17	n/d
CC2	aug-cc-pVTZ	1.358	1.466	120.58	118.85	n/d
CC2	cc-pVQZ	1.355	1.464	120.41	119.18	n/d
CC2	aug-cc-pVQZ	1.356	1.464	120.40	119.20	n/d
DFT(B3LYP)	6-31G(d,p)	1.347	1.476	120.53	118.95	2 [-543, -81 cm^{-1}]
DFT(B3LYP)	cc-pVDZ	1.350	1.477	120.51	118.98	2 [-529, -65 cm^{-1}]
DFT(B3LYP)	cc-pVTZ	1.341	1.471	120.51	118.97	2 [-462, -85 cm^{-1}]
DFT(B3LYP)	cc-pVQZ	1.341	1.470	120.51	118.98	2 [-458, -89 cm^{-1}]
DFT(M06-2X)	6-31G(d,p)	1.341	1.474	120.58	118.84	1 [-98 cm^{-1}]
DFT(M06-2X)	cc-pVDZ	1.344	1.475	120.55	118.90	1 [-94 cm^{-1}]
DFT(M06-2X)	cc-pVTZ	1.336	1.470	120.55	118.89	1 [-96 cm^{-1}]
DFT(M06-2X)	cc-pVQZ	1.335	1.470	120.55	118.90	1 [-90 cm^{-1}]
DFT(CAM-B3LYP)	6-31G(d,p)	1.339	1.472	120.54	118.91	2 [-636, -78 cm^{-1}]
DFT(CAM-B3LYP)	cc-pVDZ	1.342	1.474	120.52	118.95	2 [-620, -63 cm^{-1}]
DFT(CAM-B3LYP)	cc-pVTZ	1.333	1.467	120.53	118.95	2 [-523, -82 cm^{-1}]
DFT(CAM-B3LYP)	cc-pVQZ	1.333	1.467	120.55	118.90	2 [-516, -86 cm^{-1}]

n/d - *not determined (because of computational cost or some numerical troubles)*

Table S7. Selected bond lengths [Å] and valence bond angles [°] between carbon atoms in antiquinoid-like neutral variant of benzene ring in the first electronic triplet excided state obtained from chosen approaches of quantum chemistry computational methods. C-C bond are labelled by designations introduced by **Figure 1**. If it is indicated, vibrational analysis was carried out for the equilibrium structure. All obtained structures are flat, possible imaginary frequencies relate to the swing of hydrogen atoms off the plane of symmetry, or are numerical artefacts of DFT methodology.

Total charge: 0

Spin multiplicity: 1

Geometry optimization state: T_1

BZ(AQ) R^0_{T1}

Method	Basis set	Bond lenghts [Å]		Valence bond angles [°]		Imag. freq.
		a^0_{T1}	b^0_{T1}	∠(a^0_{T1}, b^0_{T1})	∠(b^0_{T1}, b^0_{T1})	
CASPT2 (6e,6o), 0-IPEA	6-31G(d,p)	1.487	1.403	119.63	120.74	n/d
CASPT2 (6e,6o), 0-IPEA	cc-pVDZ	1.496	1.412	119.63	120.73	n/d
CASPT2 (6e,6o), 0-IPEA	cc-pVTZ	1.491	1.397	119.62	120.76	n/d
CASPT2 (6e,6o), S-IPEA	6-31G(d,p)	1.497	1.398	119.57	120.87	n/d
CASPT2 (6e,6o), S-IPEA	cc-pVDZ	1.507	1.406	119.56	120.87	n/d
CASPT2 (6e,6o), S-IPEA	cc-pVTZ	1.499	1.392	119.54	120.92	n/d
CASSCF (6e,6o)	6-31G(d,p)	1.499	1.398	119.53	120.93	n/d
CASSCF (6e,6o)	cc-pVDZ	1.501	1.400	119.54	120.93	n/d
CASSCF (6e,6o)	cc-pVTZ	1.500	1.392	119.52	120.97	n/d
CASSCF (6e,6o)	cc-pVQZ	1.499	1.391	119.52	120.96	n/d
CC2	cc-pVDZ	1.511	1.407	119.52	120.96	n/d
CC2	aug-cc-pVDZ	1.513	1.408	119.52	120.96	n/d
CC2	cc-pVTZ	1.505	1.393	119.49	121.02	n/d
CC2	aug-cc-pVTZ	1.505	1.393	119.50	120.98	n/d
CC2	cc-pVQZ	1.503	1.390	119.49	121.01	n/d
CC2	aug-cc-pVQZ	1.503	1.391	119.51	120.98	n/d
DFT(B3LYP)	6-31G(d,p)	1.522	1.389	119.40	121.19	0
DFT(B3LYP)	cc-pVDZ	1.523	1.391	119.41	121.17	0
DFT(B3LYP)	cc-pVTZ	1.517	1.383	119.40	121.19	0
DFT(B3LYP)	cc-pVQZ	1.517	1.382	119.41	121.18	0
DFT(M06-2X)	6-31G(d,p)	1.518	1.384	119.43	121.14	1 [-1199 cm-1]
DFT(M06-2X)	cc-pVDZ	1.519	1.387	119.44	121.11	1 [-1166cm-1]
DFT(M06-2X)	cc-pVTZ	1.515	1.380	119.44	121.12	1 [-1060cm-1]
DFT(M06-2X)	cc-pVQZ	1.514	1.379	119.45	121.10	1 [-1131cm-1]
DFT(CAM-B3LYP)	6-31G(d,p)	1.518	1.383	119.43	121.14	0
DFT(CAM-B3LYP)	cc-pVDZ	1.519	1.386	119.44	121.12	0
DFT(CAM-B3LYP)	cc-pVTZ	1.513	1.377	119.43	121.13	0
DFT(CAM-B3LYP)	cc-pVQZ	1.513	1.377	119.44	121.18s	0

n/d - *not determined (because of computational cost or some numerical troubles)*

Table S8. Grochala, Albrecht, and Hoffmann Bond Length Rule formula values for selected carbon-carbon bonds [Å] and valence bond angles [°] in quinoid-like benzene variants. C-C bonds are labelled by designations introduced by **Figure 1**. The GAH rule is fulfilled more precisely when the value calculated according the formula is closer to zero. The charge of the molecule and its spin multiplicity can affect each bond length. Therefore unsigned relative percentage values are given in brackets. They were calculated by dividing the absolute value between the difference in length of a given bond in its longest and shortest form.

BZ(Q) ΔGAH(R)

Method	Basis set	$\Delta GAH(R) = R^{+} + R^{-} - R^{0} - R^{0}_{T1}$					
		a[Å]		b[Å]		∠(a⁰, b⁰) [°]	∠(b⁰, b⁰) [°]
CASPT2, 0-IPEA	6-31G(d,p)	-0.014	(55.8%)	0.010	(16.0%)	0.45	-0.93
CASPT2, 0-IPEA	cc-pVDZ	-0.013	(51.8%)	0.010	(16.2%)	0.42	-0.83
CASPT2, 0-IPEA	cc-pVTZ	-0.009	(31.0%)	0.008	(12.5%)	0.36	-0.72
CASPT2, S-IPEA	6-31G(d,p)	-0.007	(22.2%)	0.006	(8.3%)	0.39	-0.77
CASPT2, S-IPEA	cc-pVDZ	-0.006	(17.9%)	0.006	(8.2%)	0.35	-0.68
CASPT2, 0-IPEA	cc-pVTZ	-0.003	(8.6%)	0.005	(7.1%)	0.28	-0.57
CASSCF	6-31G(d,p)	-0.012	(32.9%)	-0.002	(3.4%)	0.38	-0.75
CASSCF	cc-pVDZ	-0.011	(29.7%)	-0.003	(3.8%)	0.33	-0.67
CASSCF	cc-pVTZ	-0.008	(22.1%)	-0.004	(5.2%)	0.28	-0.56
CASSCF	cc-pVQZ	-0.008	(20.3%)	-0.005	(6.5%)	0.25	-0.49
CC2	cc-pVDZ	-0.016	(47.3%)	-0.011	(15.6%)	5.25	-10.39
CC2	aug-cc-pVDZ	-0.013	(36.7%)	-0.014	(20.9%)	5.10	-10.19
CC2	cc-pVTZ	-0.013	(35.9%)	-0.013	(18.4%)	5.21	-10.44
CC2	aug-cc-pVTZ	-0.011	(28.6%)	-0.017	(24.1%)	4.89	-9.79
CC2	cc-pVQZ	-0.013	(33.7%)	-0.014	(20.2%)	5.18	-10.37
CC2	aug-cc-pVQZ	-0.010	(27.3%)	-0.017	(24.6%)	5.02	-10.03
DFT(B3LYP)	6-31G(d,p)	0.007	(13.8%)	-0.001	(1.1%)	0.18	-0.36
DFT(B3LYP)	cc-pVDZ	0.006	(13.3%)	-0.001	(1.4%)	0.15	-0.31
DFT(B3LYP)	cc-pVTZ	0.007	(13.6%)	-0.001	(1.8%)	0.12	-0.24
DFT(B3LYP)	cc-pVQZ	0.007	(14.0%)	-0.002	(2.1%)	0.10	-0.21
DFT(M06-2X)	6-31G(d,p)	0.006	(11.8%)	-0.002	(2.8%)	0.12	-0.24
DFT(M06-2X)	cc-pVDZ	0.007	(12.7%)	-0.002	(2.9%)	0.09	-0.18
DFT(M06-2X)	cc-pVTZ	0.006	(11.6%)	-0.003	(3.2%)	0.06	-0.11
DFT(M06-2X)	cc-pVQZ	0.007	(12.6%)	-0.003	(3.3%)	0.04	-0.08
DFT(CAM-B3LYP)	6-31G(d,p)	0.006	(11.8%)	-0.002	(3.1%)	0.13	-0.24
DFT(CAM-B3LYP)	cc-pVDZ	0.006	(11.6%)	-0.001	(1.6%)	0.15	-0.29
DFT(CAM-B3LYP)	cc-pVTZ	0.006	(11.7%)	-0.003	(3.2%)	0.06	-0.13
DFT(CAM-B3LYP)	cc-pVQZ	0.006	(12.3%)	-0.003	(3.8%)	0.02	-0.04

Table S9. Grochala, Albrecht, and Hoffmann Bond Length Rule formula values for selected carbon-carbon bonds [Å] and valence bond angles [°] in anti-quinoid-like benzene variants. C-C bonds are labelled by designations introduced by **Figure 1**. The GAH rule is fulfilled more precisely when the value calculated according the formula is closer to zero. The charge of the molecule and its spin multiplicity can affect each bond length. Therefore, absolute values of relative percentage values are given in brackets. They were calculated by dividing the absolute value between the difference in length of a given bond in its longest and shortest form. TBD – to be determined.

BZ(AQ) ΔGAH(R)

Method	Basis set	$\Delta GAH(R) = R^{+} + R^{-} - R^{0} - R^{0}_{T1}$					
		a[Å]		a[Å]		∠(a⁰, b⁰) [°]	∠(b⁰, b⁰) [°]
CASPT2, 0-IPEA	6-31G(d,p)	0.026	(29.1%)	-0.010	(90.9%)	-0.36	0.73
CASPT2, 0-IPEA	cc-pVDZ	0.025	(28.4%)	-0.009	(84.5%)	-0.34	0.68
CASPT2, 0-IPEA	cc-pVTZ	0.019	(19.8%)	-0.007	(87.5%)	-1.92	3.83
CASPT2, S-IPEA	6-31G(d,p)	0.015	(14.9%)	-0.005	(83.9%)	-0.30	0.59
CASPT2, S-IPEA	cc-pVDZ	0.014	(13.4%)	-0.010	(100.0%)	-0.26	0.51
CASPT2, S-IPEA	cc-pVTZ	0.011	(10.4%)	-0.003	(37.5%)	-1.86	3.71
CASSCF	6-31G(d,p)	0.006	(5.7%)	-0.011	(122.8%)	-0.27	0.54
CASSCF	cc-pVDZ	0.005	(4.4%)	-0.008	(92.6%)	-0.24	0.45
CASSCF	cc-pVTZ	0.002	(2.0%)	-0.008	(101.2%)	-0.17	0.34
CASSCF	cc-pVQZ	0.001	(1.0%)	-0.008	(93.1%)	-0.14	0.29
CC2	cc-pVDZ	0.003	(2.4%)	-0.020	(92.9%)	-4.92	9.83
CC2	aug-cc-pVDZ	-0.005	(4.7%)	-0.019	(74.4%)	-4.75	9.52
CC2	cc-pVTZ	-0.002	(2.0%)	-0.019	(78.3%)	-4.92	9.85
CC2	aug-cc-pVTZ	-0.009	(8.6%)	-0.018	(69.3%)	-4.75	9.53
CC2	cc-pVQZ	-0.004	(4.1%)	-0.018	(74.3%)	-4.89	9.78
CC2	aug-cc-pVQZ	-0.012	(11.0%)	-0.017	(67.7%)	-4.69	9.39
DFT(B3LYP)	6-31G(d,p)	-0.002	(1.7%)	0.003	(31.0%)	-0.11	0.22
DFT(B3LYP)	cc-pVDZ	-0.002	(1.9%)	0.003	(29.2%)	-0.08	0.17
DFT(B3LYP)	cc-pVTZ	-0.003	(2.5%)	0.003	(28.6%)	-0.05	0.09
DFT(B3LYP)	cc-pVQZ	-0.004	(3.2%)	0.003	(28.7%)	-0.03	-0.25
DFT(M06-2X)	6-31G(d,p)	-0.002	(1.4%)	0.002	(18.8%)	-0.09	0.17
DFT(M06-2X)	cc-pVDZ	-0.002	(1.7%)	0.002	(18.6%)	-0.05	0.10
DFT(M06-2X)	cc-pVTZ	-0.002	(2.0%)	0.002	(17.0%)	-0.01	0.01
DFT(M06-2X)	cc-pVQZ	-0.003	(2.5%)	0.002	(19.6%)	0.01	-0.03
DFT(CAM-B3LYP)	6-31G(d,p)	-0.003	(2.4%)	0.001	(9.3%)	-0.07	0.14
DFT(CAM-B3LYP)	cc-pVDZ	-0.002	(1.9%)	0.001	(16.3%)	-0.06	0.13
DFT(CAM-B3LYP)	cc-pVTZ	-0.004	(3.1%)	0.001	(10.1%)	0.00	0.01
DFT(CAM-B3LYP)	cc-pVQZ	-0.005	(3.5%)	0.001	(12.8%)	0.01	-0.09

Table S10. Selected bond lengths [Å] between carbon atoms in anionic variant of cyclobutadiene (CBDE) ring obtained from chosen approaches of quantum chemistry computational methods. C-C bond are labelled by designations introduced by **Figure 1**. If it is indicated, vibrational analysis was carried out for the equilibrium structure. All obtained structures are flat, possible imaginary frequencies relate to the swing of hydrogen atoms off the plane of symmetry, or are numerical artefacts of DFT methodology. All internal angles in carbon ring are right.

Total charge: -1

Spin multiplicity: 2

Geometry optimization state: D_0

CBDE R$^-$

Method	Basis set	Bond lenghts [Å]		Imag. freq.
		a^-	b^-	
CASPT2 (5e,4o), 0-IPEA	6-31G(d,p)	1.510	1.401	n/d
CASPT2 (5e,4o), 0-IPEA	cc-pVDZ	1.524	1.413	n/d
CASPT2 (5e,4o), 0-IPEA	cc-pVTZ	1.514	1.401	n/d
CASPT2 (5e,4o), S-IPEA	6-31G(d,p)	1.509	1.398	n/d
CASPT2 (5e,4o), S-IPEA	cc-pVDZ	1.522	1.418	n/d
CASPT2 (5e,4o), S-IPEA	cc-pVTZ	1.512	1.398	n/d
CASSCF (5e,4o)	6-31G(d,p)	1.500	1.393	3 [-544, -397, -166 cm^{-1}]
CASSCF (5e,4o)	cc-pVDZ	1.505	1.398	3 [-495, -300, -265 cm^{-1}]
CASSCF (5e,4o)	cc-pVTZ	1.500	1.390	2 [-489, -290 cm^{-1}]
CASSCF (5e,4o)	cc-pVQZ	1.499	1.390	3 [-506, -337, -106 cm^{-1}]
CC2	cc-pVDZ	1.521	1.413	n/d
CC2	aug-cc-pVDZ	1.523	1.416	n/d
CC2	cc-pVTZ	1.507	1.396	n/d
CC2	aug-cc-pVTZ	1.506	1.396	n/d
CC2	cc-pVQZ	1.505	1.395	n/d
CC2	aug-cc-pVQZ	1.504	1.395	n/d
DFT(B3LYP)	6-31G(d,p)	1.516	1.394	2 [-543, -374 cm^{-1}]
DFT(B3LYP)	cc-pVDZ	1.520	1.399	2 [-467, -198 cm^{-1}]
DFT(B3LYP)	cc-pVTZ	1.513	1.390	2 [-477, -191 cm^{-1}]
DFT(B3LYP)	cc-pVQZ	1.512	1.390	2 [-508, -301 cm^{-1}]
DFT(M06-2X)	6-31G(d,p)	1.506	1.394	2 [-473, -188 cm^{-1}]
DFT(M06-2X)	cc-pVDZ	1.510	1.392	1 [-391 cm^{-1}]
DFT(M06-2X)	cc-pVTZ	1.506	1.385	1 [-369 cm^{-1}]
DFT(M06-2X)	cc-pVQZ	1.505	1.385	1 [-395 cm^{-1}]
DFT(CAM-B3LYP)	6-31G(d,p)	1.508	1.379	2 [-486, -235 cm^{-1}]
DFT(CAM-B3LYP)	cc-pVDZ	1.512	1.392	1 [-411 cm^{-1}]
DFT(CAM-B3LYP)	cc-pVTZ	1.505	1.384	1 [-421 cm^{-1}]
DFT(CAM-B3LYP)	cc-pVQZ	1.504	1.384	2 [-451, -89 cm^{-1}]

n/d - *not determined (because of computational cost or some numerical troubles)*

Table S11. Selected bonds lengths [Å] between carbon atoms in cationic variant of cyclobutadiene (CBDE) ring obtained from chosen approaches of quantum chemistry computational methods C-C bond are labelled by designations introduced by **Figure 1**. If it is indicated, vibrational analysis was carried out for the equilibrium structure. All obtained structures are flat. All internal angles in carbon ring are right.

Total charge: 1

Spin multiplicity: 2

Geometry optimization state: D_0

CBDE R$^+$

Method	Basis set	Bond lenghts [Å]		Imag. freq.
		a^+	b^+	
CASPT2 (3e,4o), 0-IPEA	6-31G(d,p)	1.502	1.385	n/d
CASPT2 (3e,4o), 0-IPEA	cc-pVDZ	1.514	1.396	n/d
CASPT2 (3e,4o), 0-IPEA	cc-pVTZ	1.502	1.382	n/d
CASPT2 (3e,4o), S-IPEA	6-31G(d,p)	1.500	1.383	n/d
CASPT2 (3e,4o), S-IPEA	cc-pVDZ	1.511	1.394	n/d
CASPT2 (3e,4o), S-IPEA	cc-pVTZ	1.500	1.380	n/d
CASSCF (3e,4o)	6-31G(d,p)	1.485	1.378	0
CASSCF (3e,4o)	cc-pVDZ	1.488	1.382	0
CASSCF (3e,4o)	cc-pVTZ	1.483	1.375	0
CASSCF (3e,4o)	cc-pVQZ	1.482	1.374	0
CC2	cc-pVDZ	1.510	1.398	n/d
CC2	aug-cc-pVDZ	1.512	1.399	n/d
CC2	cc-pVTZ	1.494	1.380	n/d
CC2	aug-cc-pVTZ	1.494	1.380	n/d
CC2	cc-pVQZ	1.492	1.378	n/d
CC2	aug-cc-pVQZ	1.492	1.378	n/d
DFT(B3LYP)	6-31G(d,p)	1.505	1.380	0
DFT(B3LYP)	cc-pVDZ	1.508	1.383	0
DFT(B3LYP)	cc-pVTZ	1.501	1.373	0
DFT(B3LYP)	cc-pVQZ	1.500	1.373	0
DFT(M06-2X)	6-31G(d,p)	1.497	1.375	0
DFT(M06-2X)	cc-pVDZ	1.499	1.378	0
DFT(M06-2X)	cc-pVTZ	1.494	1.370	0
DFT(M06-2X)	cc-pVQZ	1.494	1.369	0
DFT(CAM-B3LYP)	6-31G(d,p)	1.497	1.374	0
DFT(CAM-B3LYP)	cc-pVDZ	1.500	1.377	0
DFT(CAM-B3LYP)	cc-pVTZ	1.493	1.368	0
DFT(CAM-B3LYP)	cc-pVQZ	1.493	1.367	0

n/d - *not determined (because of computational cost or some numerical troubles)*

Table S12. Selected bonds lengths [Å] between carbon atoms in neutral variant of cyclobutadiene (CBDE) ring obtained from chosen approaches of quantum chemistry computational methods. C-C bond are labelled by designations introduced by **Figure 1**. If it is indicated, vibrational analysis was carried out for the equilibrium structure. All obtained structures are flat. All internal angles in carbon ring are right.

Total charge: 0
Spin multiplicity: 1
Geometry optimization state: S_0

CBDE R^0

Method	Basis set	Bond lenghts [Å] a^0	b^0	Imag. freq.
CASPT2 (4e,4o), 0-IPEA	6-31G(d,p)	1.547	1.360	n/d
CASPT2 (4e,4o), 0-IPEA	cc-pVDZ	1.560	1.371	n/d
CASPT2 (4e,4o), 0-IPEA	cc-pVTZ	1.552	1.355	n/d
CASPT2 (4e,4o), S-IPEA	6-31G(d,p)	1.552	1.354	n/d
CASPT2 (4e,4o), S-IPEA	cc-pVDZ	1.566	1.365	n/d
CASPT2 (4e,4o), S-IPEA	cc-pVTZ	1.557	1.350	n/d
CASSCF (4e,4o)	6-31G(d,p)	1.545	1.353	0
CASSCF (4e,4o)	cc-pVDZ	1.550	1.357	0
CASSCF (4e,4o)	cc-pVTZ	1.548	1.347	0
CASSCF (4e,4o)	cc-pVQZ	1.547	1.346	0
CC2	cc-pVDZ	1.578	1.360	n/d
CC2	aug-cc-pVDZ	1.578	1.362	n/d
CC2	cc-pVTZ	1.563	1.342	n/d
CC2	aug-cc-pVTZ	1.562	1.342	n/d
CC2	cc-pVQZ	1.561	1.340	n/d
CC2	aug-cc-pVQZ	1.560	1.340	n/d
DFT(B3LYP)	6-31G(d,p)	1.578	1.335	0
DFT(B3LYP)	cc-pVDZ	1.581	1.339	0
DFT(B3LYP)	cc-pVTZ	1.575	1.329	0
DFT(B3LYP)	cc-pVQZ	1.574	1.329	0
DFT(M06-2X)	6-31G(d,p)	1.569	1.330	0
DFT(M06-2X)	cc-pVDZ	1.572	1.334	0
DFT(M06-2X)	cc-pVTZ	1.567	1.325	0
DFT(M06-2X)	cc-pVQZ	1.566	1.325	0
DFT(CAM-B3LYP)	6-31G(d,p)	1.570	1.329	0
DFT(CAM-B3LYP)	cc-pVDZ	1.573	1.333	0
DFT(CAM-B3LYP)	cc-pVTZ	1.567	1.323	0
DFT(CAM-B3LYP)	cc-pVQZ	1.566	1.323	0

n/d - *not determined (because of computational cost or some numerical troubles)*

Table S13. Selected bonds lengths [Å] between carbon atoms in neutral variant of cyclobutadiene (CBDE) ring in firs electronic triplet excited state obtained from chosen approaches of quantum chemistry computational methods. C-C bond are labelled by designations introduced by **Figure 1**. If it is indicated, vibrational analysis was carried out for the equilibrium structure. All obtained structures are flat. All internal angles in carbon ring are right.

Total charge: 0

Spin multiplicity: 1

Geometry optimization state: T_1

CBDE $R^0{}_{T1}$

Method	Basis set	Bond lenght [Å] $a^0{}_{T1} = b^0{}_{T1}$	Imag. freq.
CASPT2 (4e,4o), 0-IPEA	6-31G(d,p)	1.441	n/d
CASPT2 (4e,4o), 0-IPEA	cc-pVDZ	1.453	n/d
CASPT2 (4e,4o), 0-IPEA	cc-pVTZ	1.439	n/d
CASPT2 (4e,4o), S-IPEA	6-31G(d,p)	1.440	n/d
CASPT2 (4e,4o), S-IPEA	cc-pVDZ	1.452	n/d
CASPT2 (4e,4o), S-IPEA	cc-pVTZ	1.438	n/d
CASSCF (4e,4o)	6-31G(d,p)	1.439	0
CASSCF (4e,4o)	cc-pVDZ	1.442	0
CASSCF (4e,4o)	cc-pVTZ	1.436	0
CASSCF (4e,4o)	cc-pVQZ	1.435	0
CC2	cc-pVDZ	1.453	n/d
CC2	aug-cc-pVDZ	1.455	n/d
CC2	cc-pVTZ	1.436	n/d
CC2	aug-cc-pVTZ	1.437	n/d
CC2	cc-pVQZ	1.435	n/d
CC2	aug-cc-pVQZ	1.435	n/d
DFT(B3LYP)	6-31G(d,p)	1.441	0
DFT(B3LYP)	cc-pVDZ	1.444	0
DFT(B3LYP)	cc-pVTZ	1.436	0
DFT(B3LYP)	cc-pVQZ	1.436	0
DFT(M06-2X)	6-31G(d,p)	1.433	0
DFT(M06-2X)	cc-pVDZ	1.437	0
DFT(M06-2X)	cc-pVTZ	1.431	0
DFT(M06-2X)	cc-pVQZ	1.430	0
DFT(CAM-B3LYP)	6-31G(d,p)	1.434	0
DFT(CAM-B3LYP)	cc-pVDZ	1.438	0
DFT(CAM-B3LYP)	cc-pVTZ	1.430	0
DFT(CAM-B3LYP)	cc-pVQZ	1.429	0

n/d - *not determined (because of computational cost or some numerical troubles)*

Table S14. Grochala, Albrecht, and Hoffmann Bond Length Rule formula values [Å] for carbon-carbon bonds in cyclobutadiene variants. C-C bonds are labelled by designations introduced by **Figure 1**. The GAH rule is fulfilled more precisely when the value calculated according the formula is closer to zero. The charge of the molecule and its spin multiplicity can affect each bond length. Therefore unsigned relative percentage values are given in brackets. They were calculated by dividing the absolute value between the difference in length of a given bond in its longest and shortest form.

CBDE ΔGAH(R)

Method	Basis set	$\Delta GAH(R) = R^+ + R^- - R^0 - R^0_{T1}$ [Å]			
		a		b	
CASPT2, 0-IPEA	6-31G(d,p)	0.024	(22.6%)	-0.015	(18.5%)
CASPT2, 0-IPEA	cc-pVDZ	0.025	(23.4%)	-0.015	(18.3%)
CASPT2, 0-IPEA	cc-pVTZ	0.025	(22.1%)	-0.011	(13.1%)
CASPT2, S-IPEA	6-31G(d,p)	0.017	(15.2%)	-0.013	(15.1%)
CASPT2, S-IPEA	cc-pVDZ	0.015	(13.2%)	-0.005	(5.7%)
CASPT2, S-IPEA	cc-pVTZ	0.017	(14.3%)	-0.010	(11.4%)
CASSCF	6-31G(d,p)	0.001	(0.9%)	-0.021	(22.4%)
CASSCF	cc-pVDZ	0.001	(0.9%)	-0.019	(22.4%)
CASSCF	cc-pVTZ	-0.001	(0.9%)	-0.019	(20.2%)
CASSCF	cc-pVQZ	-0.001	(0.9%)	-0.017	(19.1%)
CC2	cc-pVDZ	0.001	(0.8%)	-0.001	(1.4%)
CC2	aug-cc-pVDZ	0.001	(1.1%)	-0.002	(1.9%)
CC2	cc-pVTZ	0.002	(1.2%)	-0.002	(1.7%)
CC2	aug-cc-pVTZ	0.002	(1.6%)	-0.003	(2.8%)
CC2	cc-pVQZ	0.002	(1.4%)	-0.001	(1.4%)
CC2	aug-cc-pVQZ	0.002	(1.3%)	-0.002	(2.4%)
DFT(B3LYP)	6-31G(d,p)	0.002	(1.5%)	-0.002	(1.9%)
DFT(B3LYP)	cc-pVDZ	0.003	(2.2%)	-0.001	(1.0%)
DFT(B3LYP)	cc-pVTZ	0.003	(2.2%)	-0.002	(1.9%)
DFT(B3LYP)	cc-pVQZ	0.002	(1.4%)	-0.002	(1.9%)
DFT(M06-2X)	6-31G(d,p)	0.001	(0.7%)	0.006	(5.8%)
DFT(M06-2X)	cc-pVDZ	0.000	(0.0%)	-0.001	(1.0%)
DFT(M06-2X)	cc-pVTZ	0.002	(1.5%)	-0.001	(0.9%)
DFT(M06-2X)	cc-pVQZ	0.003	(2.2%)	-0.001	(1.0%)
DFT(CAM-B3LYP)	6-31G(d,p)	0.001	(0.7%)	-0.010	(9.5%)
DFT(CAM-B3LYP)	cc-pVDZ	0.001	(0.7%)	-0.002	(1.9%)
DFT(CAM-B3LYP)	cc-pVTZ	0.001	(0.7%)	-0.001	(0.9%)
DFT(CAM-B3LYP)	cc-pVQZ	0.002	(1.5%)	-0.001	(0.9%)

Table S15. Bonds lengths [Å] between carbon atoms in neutral cyclopropenium radical (CP) obtained from selected methods of computational quantum chemistry. Vibrational analysis was carried out for the equilibrium structure

Total charge: **0**
Spin multiplicity: **2**
Geometry optimization state: **D_0**

Method	Basis set	Bond lenghts [Å]			Imag. freq.
		C_1–C_2	C_2–C_3	C_1–C_3	
DFT(B3LYP)	6-31G(d,p)	1.465	1.465	1.315	0
DFT(B3LYP)	cc-pVDZ	1.469	1.469	1.320	0
DFT(B3LYP)	cc-pVTZ	1.456	1.456	1.308	0
DFT(B3LYP)	cc-pVQZ	1.455	1.455	1.307	0
DFT(M06-2X)	6-31G(d,p)	1.458	1.458	1.310	0
DFT(M06-2X)	cc-pVDZ	1.464	1.464	1.315	0
DFT(M06-2X)	cc-pVTZ	1.453	1.453	1.304	0
DFT(M06-2X)	cc-pVQZ	1.452	1.452	1.304	0
DFT(CAM-B3LYP)	6-31G(d,p)	1.460	1.460	1.310	0
DFT(CAM-B3LYP)	cc-pVDZ	1.463	1.463	1.314	0
DFT(CAM-B3LYP)	cc-pVTZ	1.450	1.450	1.302	0
DFT(CAM-B3LYP)	cc-pVQZ	1.448	1.448	1.301	0

Table S16. Bonds lengths [Å] between carbon atoms in cyclopropenium radical bication (CP^{2+}) obtained from selected methods of computational quantum chemistry. Vibrational analysis was carried out for the equilibrium structure.

Total charge: **2**
Spin multiplicity: **2**
Geometry optimization state: **D_0**

Method	Basis set	Bond lenghts [Å]			Imag. freq.
		C_1–C_2	C_2–C_3	C_1–C_3	
DFT(B3LYP)	6-31G(d,p)	1.343	1.343	1.600	0
DFT(B3LYP)	cc-pVDZ	1.348	1.348	1.607	0
DFT(B3LYP)	cc-pVTZ	1.336	1.336	1.599	0
DFT(B3LYP)	cc-pVQZ	1.335	1.335	1.597	0
DFT(M06-2X)	6-31G(d,p)	1.341	1.341	1.572	0
DFT(M06-2X)	cc-pVDZ	1.347	1.347	1.607	0
DFT(M06-2X)	cc-pVTZ	1.335	1.335	1.572	0
DFT(M06-2X)	cc-pVQZ	1.335	1.335	1.570	0
DFT(CAM-B3LYP)	6-31G(d,p)	1.338	1.338	1.579	0
DFT(CAM-B3LYP)	cc-pVDZ	1.343	1.343	1.588	0
DFT(CAM-B3LYP)	cc-pVTZ	1.331	1.331	1.579	0
DFT(CAM-B3LYP)	cc-pVQZ	1.330	1.330	1.577	0

Table S17. Bonds lengths [Å] between carbon atoms in cyclopropenium cation (CP^+) in the ground singlet state obtained from selected methods of computational quantum chemistry. Vibrational analysis was carried out for the equilibrium structure.

Total charge: 1

Spin multiplicity: 1

Geometry optimization state: S_0

Method	Basis set	Bond lenghts [Å]			Imag. freq.
		C_1–C_2	C_2–C_3	C_1–C_3	
DFT(B3LYP)	6-31G(d,p)	1.366	1.366	1.366	0
DFT(B3LYP)	cc-pVDZ	1.371	1.371	1.371	0
DFT(B3LYP)	cc-pVTZ	1.358	1.358	1.358	0
DFT(B3LYP)	cc-pVQZ	1.357	1.357	1.357	0
DFT(M06-2X)	6-31G(d,p)	1.362	1.362	1.362	0
DFT(M06-2X)	cc-pVDZ	1.367	1.367	1.367	0
DFT(M06-2X)	cc-pVTZ	1.356	1.356	1.356	0
DFT(M06-2X)	cc-pVQZ	1.355	1.355	1.355	0
DFT(CAM-B3LYP)	6-31G(d,p)	1.361	1.361	1.361	0
DFT(CAM-B3LYP)	cc-pVDZ	1.366	1.366	1.366	0
DFT(CAM-B3LYP)	cc-pVTZ	1.352	1.352	1.352	0
DFT(CAM-B3LYP)	cc-pVQZ	1.351	1.351	1.351	0

Table S18. Bonds lengths [Å] between carbon atoms in cyclopropenium cation (CP^+) in the first excited triplet state obtained from selected methods of computational quantum chemistry. Vibrational analysis was carried out for the equilibrium structure.

Total charge: 1

Spin multiplicity: 3

Geometry optimization state: T_1

Method	Basis set	Bond lenghts [Å]			Imag. freq.
		C_1–C_2	C_2–C_3	C_1–C_3	
DFT(B3LYP)	6-31G(d,p)	1.322	1.370	1.956	0
DFT(B3LYP)	cc-pVDZ	1.327	1.368	1.968	0
DFT(B3LYP)	cc-pVTZ	1.304	1.359	1.958	0
DFT(B3LYP)	cc-pVQZ	1.303	1.359	1.955	0
DFT(M06-2X)	6-31G(d,p)	1.298	1.405	1.874	0
DFT(M06-2X)	cc-pVDZ	1.302	1.405	1.880	0
DFT(M06-2X)	cc-pVTZ	1.291	1.397	1.872	0
DFT(M06-2X)	cc-pVQZ	1.289	1.399	1.868	0
DFT(CAM-B3LYP)	6-31G(d,p)	1.310	1.374	1.892	0
DFT(CAM-B3LYP)	cc-pVDZ	1.316	1.376	1.903	0
DFT(CAM-B3LYP)	cc-pVTZ	1.304	1.365	1.894	0
DFT(CAM-B3LYP)	cc-pVQZ	1.303	1.365	1.890	0

Table S19. Mulliken atomic spin densities obtained with chosen computational approaches for quinoid isomer of anionic form of benzene molecule. In the right part of the table atomic spin densities of hydrogens are summed into heavy atoms they are connected to. Please note, that data for CASPT2 method are computed for equilibrium geometries obtained at this level of theory, however spin densities are computed basing on CASSCF wavefunction.

BZ(Q) R^{-}

Method	Basis set	H1	C2	C3	H4	C5	H6	C7	H8	C9	H10	C11	H12	H1+C2	C3+H4	C5+H6	C7+H8	C9+J10	C11+H12
CASPT2, 0-IPEA	6-31G(d,p)	0.0021	0.4269	0.0352	0.0003	0.0352	0.0003	0.4269	0.0021	0.0352	0.0003	0.0352	0.0003	0.4290	0.0355	0.0355	0.4290	0.0355	0.0355
CASPT2, 0-IPEA	cc-pVDZ	0.0040	0.4205	0.0372	0.0005	0.0372	0.0005	0.4205	0.0040	0.0372	0.0005	0.0372	0.0005	0.4245	0.0377	0.0377	0.4245	0.0377	0.0377
CASPT2, 0-IPEA	cc-pVTZ	0.0089	0.4074	0.0406	0.0012	0.0406	0.0012	0.4074	0.0089	0.0406	0.0012	0.0406	0.0012	0.4163	0.0418	0.0418	0.4163	0.0418	0.0418
CASPT2, S-IPEA	6-31G(d,p)	0.0021	0.4268	0.0353	0.0003	0.0353	0.0003	0.4268	0.0021	0.0353	0.0003	0.0353	0.0003	0.4289	0.0356	0.0356	0.4289	0.0356	0.0356
CASPT2, S-IPEA	cc-pVDZ	0.0040	0.4204	0.0373	0.0005	0.0373	0.0005	0.4204	0.0040	0.0373	0.0005	0.0373	0.0005	0.4244	0.0378	0.0378	0.4244	0.0378	0.0378
CASPT2, S-IPEA	cc-pVTZ	0.0089	0.4073	0.0407	0.0012	0.0407	0.0012	0.4073	0.0089	0.0407	0.0012	0.0407	0.0012	0.4162	0.0419	0.0419	0.4162	0.0419	0.0419
CASSCF	6-31G(d,p)	0.0021	0.4269	0.0352	0.0003	0.0352	0.0003	0.4269	0.0021	0.0352	0.0003	0.0352	0.0003	0.4290	0.0355	0.0355	0.4290	0.0355	0.0355
CASSCF	cc-pVDZ	0.0041	0.4194	0.0377	0.0005	0.0377	0.0005	0.4194	0.0041	0.0377	0.0005	0.0377	0.0005	0.4235	0.0382	0.0382	0.4235	0.0382	0.0382
CASSCF	cc-pVTZ	0.0090	0.4072	0.0407	0.0012	0.0407	0.0012	0.4072	0.0090	0.0407	0.0012	0.0407	0.0012	0.4162	0.0419	0.0419	0.4162	0.0419	0.0419
CASSCF	cc-pVQZ	0.0157	0.4029	0.0383	0.0024	0.0383	0.0024	0.4029	0.0157	0.0383	0.0024	0.0383	0.0024	0.4186	0.0407	0.0407	0.4186	0.0407	0.0407
DFT(B3LYP)	6-31G(d,p)	0.0021	0.3585	0.0693	0.0004	0.0693	0.0004	0.3585	0.0021	0.0693	0.0004	0.0693	0.0004	0.3606	0.0697	0.0697	0.3606	0.0697	0.0697
DFT(B3LYP)	cc-pVDZ	0.0041	0.3517	0.0712	0.0009	0.0712	0.0009	0.3517	0.0041	0.0712	0.0009	0.0712	0.0009	0.3558	0.0721	0.0721	0.3558	0.0721	0.0721
DFT(B3LYP)	cc-pVTZ	0.0097	0.3414	0.0723	0.0021	0.0723	0.0021	0.3414	0.0097	0.0723	0.0021	0.0723	0.0021	0.3511	0.0745	0.0745	0.3511	0.0745	0.0745
DFT(B3LYP)	cc-pVQZ	0.0188	0.3368	0.0681	0.0041	0.0681	0.0041	0.3368	0.0188	0.0681	0.0041	0.0681	0.0041	0.3556	0.0722	0.0722	0.3556	0.0722	0.0722
DFT(M06-2X)	6-31G(d,p)	0.0018	0.3554	0.0710	0.0004	0.0710	0.0004	0.3554	0.0018	0.0710	0.0004	0.0710	0.0004	0.3572	0.0714	0.0714	0.3572	0.0714	0.0714
DFT(M06-2X)	cc-pVDZ	0.0037	0.3484	0.0731	0.0008	0.0731	0.0008	0.3484	0.0037	0.0731	0.0008	0.0731	0.0008	0.3522	0.0739	0.0739	0.3522	0.0739	0.0739
DFT(M06-2X)	cc-pVTZ	0.0084	0.3389	0.0745	0.0018	0.0745	0.0018	0.3389	0.0084	0.0745	0.0018	0.0745	0.0018	0.3473	0.0763	0.0763	0.3473	0.0763	0.0763
DFT(M06-2X)	cc-pVQZ	0.0141	0.3352	0.0722	0.0032	0.0722	0.0032	0.3352	0.0141	0.0722	0.0032	0.0722	0.0032	0.3493	0.0754	0.0754	0.3493	0.0754	0.0754
DFT(CAM-B3LYP)	6-31G(d,p)	0.0020	0.3575	0.0698	0.0004	0.0698	0.0004	0.3575	0.0020	0.0698	0.0004	0.0698	0.0004	0.3595	0.0702	0.0702	0.3595	0.0702	0.0702
DFT(CAM-B3LYP)	cc-pVDZ	-0.0261	0.4863	0.0232	-0.0033	0.0232	-0.0033	0.4863	-0.0261	0.0232	-0.0033	0.0232	-0.0033	0.4602	0.0199	0.0199	0.4602	0.0199	0.0199
DFT(CAM-B3LYP)	cc-pVTZ	0.0091	0.3402	0.0734	0.0020	0.0734	0.0020	0.3402	0.0091	0.0734	0.0020	0.0734	0.0020	0.3493	0.0754	0.0754	0.3493	0.0754	0.0754
DFT(CAM-B3LYP)	cc-pVQZ	0.0174	0.3361	0.0694	0.0038	0.0694	0.0038	0.3361	0.0174	0.0694	0.0038	0.0694	0.0038	0.3535	0.0732	0.0732	0.3535	0.0732	0.0732

Table S20. Mulliken atomic spin densities obtained with chosen computational approaches for antiquinoid isomer of anionic form of benzene molecule. In the right part of the table atomic spin densities of hydrogens are summed into heavy atoms they are connected to. Please note, that data for CASPT2 method are computed for equilibrium geometries obtained at this level of theory, however spin densities are computed basing on CASSCF wavefunction. TBD – to be determined.

BZ(AQ) R^-

Method	Basis set	H1	C2	C3	H4	C5	H6	C7	H8	C9	H10	C11	H12	H1+C2	C3+H4	C5+H6	C7+H8	C9+J10	C11+H12
CASPT2, 0-IPEA	6-31G(d,p)	0.0014	0.2931	-0.0888	-0.0002	0.2931	0.0014	0.2931	0.0014	-0.0888	-0.0002	0.2931	0.0014	0.2945	-0.0890	0.2945	0.2945	-0.0890	0.2945
CASPT2, 0-IPEA	cc-pVDZ	0.0027	0.2894	-0.0838	-0.0004	0.2894	0.0027	0.2894	0.0027	-0.0838	-0.0004	0.2894	0.0027	0.2921	-0.0842	0.2921	0.2921	-0.0842	0.2921
CASPT2, 0-IPEA	cc-pVTZ	0.0058	0.2842	-0.0732	-0.0008	0.2815	0.0059	0.2842	0.0059	-0.073	-0.0008	0.2842	0.0059	0.2873	-0.074	0.2874	0.2964	-0.0738	0.2864
CASPT2, S-IPEA	6-31G(d,p)	0.0014	0.2930	-0.0886	-0.0002	0.2930	0.0014	0.2930	0.0014	-0.0886	-0.0002	0.2930	0.0014	0.2944	-0.0888	0.2944	0.2944	-0.0888	0.2944
CASPT2, S-IPEA	cc-pVDZ	0.0027	0.2893	-0.0836	-0.0004	0.2893	0.0027	0.2893	0.0027	-0.0836	-0.0004	0.2893	0.0027	0.2920	-0.0840	0.2920	0.2920	-0.0840	0.2920
CASPT2, S-IPEA	cc-pVTZ	0.0059	0.2812	-0.0735	-0.0008	0.2812	0.0059	0.2812	0.0059	-0.0735	-0.0008	0.2812	0.0059	0.2871	-0.0743	0.2871	0.2871	-0.0743	0.2871
CASSCF	6-31G(d,p)	0.0014	0.2928	-0.0883	-0.0002	0.2928	0.0014	0.2928	0.0014	-0.0883	-0.0002	0.2928	0.0014	0.2942	-0.0885	0.2942	0.2942	-0.0885	0.2942
CASSCF	cc-pVDZ	0.0028	0.2885	-0.0821	-0.0004	0.2885	0.0028	0.2885	0.0028	-0.0821	-0.0004	0.2885	0.0028	0.2913	-0.0825	0.2913	0.2913	-0.0825	0.2913
CASSCF	cc-pVTZ	0.0060	0.2804	-0.0722	-0.0008	0.2804	0.0060	0.2804	0.0060	-0.0722	-0.0008	0.2804	0.0060	0.2864	-0.0730	0.2864	0.2864	-0.0730	0.2864
CASSCF	cc-pVQZ	0.0104	0.2769	-0.0738	-0.0007	0.2769	0.0104	0.2769	0.0104	-0.0738	-0.0007	0.2769	0.0104	0.2873	-0.0745	0.2873	0.2873	-0.0745	0.2873
DFT(B3LYP)	6-31G(d,p)	0.0015	0.2455	0.0061	0.0000	0.2455	0.0015	0.2455	0.0015	0.0061	0.0000	0.2455	0.0015	0.2470	0.0061	0.2470	0.2470	0.0061	0.2470
DFT(B3LYP)	cc-pVDZ	0.0029	0.2418	0.0105	0.0000	0.2418	0.0029	0.2418	0.0029	0.0105	0.0000	0.2418	0.0029	0.2448	0.0105	0.2448	0.2448	0.0105	0.2448
DFT(B3LYP)	cc-pVTZ	0.0070	0.2359	0.0142	0.0000	0.2359	0.0070	0.2359	0.0070	0.0142	0.0000	0.2359	0.0070	0.2429	0.0143	0.2429	0.2429	0.0143	0.2429
DFT(B3LYP)	cc-pVQZ	0.0135	0.2331	0.0067	0.0002	0.2331	0.0135	0.2331	0.0135	0.0067	0.0002	0.2331	0.0135	0.2465	0.0069	0.2465	0.2465	0.0069	0.2465
DFT(M06-2X)	6-31G(d,p)	0.0013	0.2454	0.0065	0.0000	0.2454	0.0013	0.2454	0.0013	0.0065	0.0000	0.2454	0.0013	0.2467	0.0065	0.2467	0.2467	0.0065	0.2467
DFT(M06-2X)	cc-pVDZ	0.0026	0.2416	0.0115	0.0000	0.2416	0.0026	0.2416	0.0026	0.0115	0.0000	0.2416	0.0026	0.2442	0.0115	0.2442	0.2442	0.0115	0.2442
DFT(M06-2X)	cc-pVTZ	0.0060	0.2357	0.0166	0.0000	0.2357	0.0060	0.2357	0.0060	0.0166	0.0000	0.2357	0.0060	0.2417	0.0167	0.2417	0.2417	0.0167	0.2417
DFT(M06-2X)	cc-pVQZ	0.0102	0.2326	0.0143	0.0001	0.2326	0.0102	0.2326	0.0102	0.0143	0.0001	0.2326	0.0102	0.2428	0.0145	0.2428	0.2428	0.0145	0.2428
DFT(CAM-B3LYP)	6-31G(d,p)	0.0014	0.2453	0.0065	0.0000	0.2453	0.0014	0.2453	0.0014	0.0065	0.0000	0.2453	0.0014	0.2467	0.0065	0.2467	0.2467	0.0065	0.2467
DFT(CAM-B3LYP)	cc-pVDZ	-0.0185	0.3304	-0.1281	0.0044	0.3304	-0.0185	0.3304	-0.0185	-0.1281	0.0044	0.3304	-0.0185	0.3118	-0.1236	0.3118	0.3118	-0.1236	0.3118
DFT(CAM-B3LYP)	cc-pVTZ	0.0065	0.2355	0.0160	0.0000	0.2355	0.0065	0.2355	0.0065	0.0160	0.0000	0.2355	0.0065	0.2420	0.0161	0.2420	0.2420	0.0161	0.2420
DFT(CAM-B3LYP)	cc-pVQZ	0.0124	0.2325	0.0100	0.0002	0.2325	0.0124	0.2325	0.0124	0.0100	0.0002	0.2325	0.0124	0.2449	0.0102	0.2449	0.2449	0.0102	0.2449

Table S21. Mulliken atomic spin densities obtained with chosen computational approaches for quinoid isomer of cationic form of benzene molecule. In the right part of the table atomic spin densities of hydrogens are summed into heavy atoms they are connected to. Please note, that data for CASPT2 method are computed for equilibrium geometries obtained at this level of theory, however spin densities are computed basing on CASSCF wavefunction.

BZ(Q) R$^+$

Method	Basis set	H1	C2	C3	H4	C5	H6	C7	H8	C9	H10	C11	H12	H1+C2	C3+H4	C5+H6	C7+H8	C9+J10	C11+H12
CASPT2, 0-IPEA	6-31G(d,p)	0.0009	0.4341	0.0318	0.0001	0.0330	0.0001	0.4341	0.0009	0.0318	0.0001	0.0330	0.0001	0.4350	0.0319	0.0331	0.4350	0.0319	0.0331
CASPT2, 0-IPEA	cc-pVDZ	0.0021	0.4289	0.0343	0.0002	0.0343	0.0002	0.4289	0.0021	0.0343	0.0002	0.0343	0.0002	0.4310	0.0345	0.0345	0.4310	0.0345	0.0345
CASPT2, 0-IPEA	cc-pVTZ	0.0044	0.4155	0.0396	0.0004	0.0396	0.0004	0.4155	0.0044	0.0396	0.0004	0.0396	0.0004	0.4199	0.0400	0.0400	0.4199	0.0400	0.0400
CASPT2, S-IPEA	6-31G(d,p)	0.0009	0.4342	0.0323	0.0001	0.0325	0.0001	0.4342	0.0009	0.0323	0.0001	0.0325	0.0001	0.4351	0.0324	0.0326	0.4351	0.0324	0.0326
CASPT2, S-IPEA	cc-pVDZ	0.0021	0.4289	0.0343	0.0002	0.0343	0.0002	0.4289	0.0021	0.0343	0.0002	0.0343	0.0002	0.4310	0.0345	0.0345	0.4310	0.0345	0.0345
CASPT2, S-IPEA	cc-pVTZ	0.0044	0.4155	0.0396	0.0004	0.0396	0.0004	0.4155	0.0044	0.0396	0.0004	0.0396	0.0004	0.4199	0.0400	0.0400	0.4199	0.0400	0.0400
CASSCF	6-31G(d,p)	0.0010	0.4340	0.0324	0.0001	0.0324	0.0001	0.4340	0.0010	0.0324	0.0001	0.0324	0.0001	0.4350	0.0325	0.0325	0.4350	0.0325	0.0325
CASSCF	cc-pVDZ	0.0022	0.4280	0.0347	0.0002	0.0347	0.0002	0.4280	0.0022	0.0347	0.0002	0.0347	0.0002	0.4302	0.0349	0.0349	0.4302	0.0349	0.0349
CASSCF	cc-pVTZ	0.0045	0.4152	0.0397	0.0004	0.0397	0.0004	0.4152	0.0045	0.0397	0.0004	0.0397	0.0004	0.4197	0.0401	0.0401	0.4197	0.0401	0.0401
CASSCF	cc-pVQZ	0.0055	0.4106	0.0414	0.0006	0.0414	0.0006	0.4106	0.0055	0.0414	0.0006	0.0414	0.0006	0.4161	0.0420	0.0420	0.4161	0.0420	0.0420
DFT(B3LYP)	6-31G(d,p)	0.0010	0.3611	0.0688	0.0002	0.0688	0.0002	0.3611	0.0010	0.0688	0.0002	0.0688	0.0002	0.3621	0.0690	0.0690	0.3621	0.0690	0.0690
DFT(B3LYP)	cc-pVDZ	0.0022	0.3565	0.0702	0.0004	0.0702	0.0004	0.3565	0.0022	0.0702	0.0004	0.0702	0.0004	0.3587	0.0707	0.0707	0.3587	0.0707	0.0707
DFT(B3LYP)	cc-pVTZ	0.0048	0.3467	0.0733	0.0009	0.0733	0.0009	0.3467	0.0048	0.0733	0.0009	0.0733	0.0009	0.3515	0.0742	0.0742	0.3515	0.0742	0.0742
DFT(B3LYP)	cc-pVQZ	0.0064	0.3430	0.0740	0.0013	0.0740	0.0013	0.3430	0.0064	0.0740	0.0013	0.0740	0.0013	0.3494	0.0753	0.0753	0.3494	0.0753	0.0753
DFT(M06-2X)	6-31G(d,p)	0.0008	0.3591	0.0699	0.0001	0.0699	0.0001	0.3591	0.0008	0.0699	0.0001	0.0699	0.0001	0.3599	0.0701	0.0701	0.3599	0.0701	0.0701
DFT(M06-2X)	cc-pVDZ	0.0018	0.3544	0.0715	0.0003	0.0715	0.0003	0.3544	0.0018	0.0715	0.0003	0.0715	0.0003	0.3562	0.0719	0.0719	0.3562	0.0719	0.0719
DFT(M06-2X)	cc-pVTZ	0.0039	0.3448	0.0749	0.0008	0.0749	0.0008	0.3448	0.0039	0.0749	0.0008	0.0749	0.0008	0.3487	0.0756	0.0756	0.3487	0.0756	0.0756
DFT(M06-2X)	cc-pVQZ	0.0046	0.3403	0.0766	0.0010	0.0766	0.0010	0.3403	0.0046	0.0766	0.0010	0.0766	0.0010	0.3449	0.0776	0.0776	0.3449	0.0776	0.0776
DFT(CAM-B3LYP)	6-31G(d,p)	0.0009	0.3608	0.0690	0.0002	0.0690	0.0002	0.3608	0.0009	0.0690	0.0002	0.0690	0.0002	0.3617	0.0691	0.0691	0.3617	0.0691	0.0691
DFT(CAM-B3LYP)	cc-pVDZ	0.0020	0.3562	0.0705	0.0004	0.0705	0.0004	0.3562	0.0020	0.0705	0.0004	0.0705	0.0004	0.3583	0.0709	0.0709	0.3583	0.0709	0.0709
DFT(CAM-B3LYP)	cc-pVTZ	0.0045	0.3464	0.0737	0.0009	0.0737	0.0009	0.3464	0.0045	0.0737	0.0009	0.0737	0.0009	0.3509	0.0745	0.0745	0.3509	0.0745	0.0745
DFT(CAM-B3LYP)	cc-pVQZ	0.0058	0.3430	0.0745	0.0011	0.0745	0.0011	0.3430	0.0058	0.0745	0.0011	0.0745	0.0011	0.3488	0.0756	0.0756	0.3488	0.0756	0.0756

Table S22. Mulliken atomic spin densities obtained with chosen computational approaches for antiquinoid isomer of cationic form of benzene molecule. In the right part of the table atomic spin densities of hydrogens are summed into heavy atoms they are connected to. Please note, that data for CASPT2 method are computed for equilibrium geometries obtained at this level of theory, however spin densities are computed basing on CASSCF wavefunction.

BZ(AQ) R+

Method	Basis set	H1	C2	C3	H4	C5	H6	C7	H8	C9	H10	C11	H12	H1+C2	C3+H4	C5+H6	C7+H8	C9+J10	C11+H12
CASPT2, 0-IPEA	6-31G(d,p)	0.0006	0.2986	-0.0990	-0.0002	0.2993	0.0006	0.2986	0.0006	-0.0990	-0.0002	0.2993	0.0006	0.2992	-0.0992	0.2999	0.2992	-0.0992	0.2999
CASPT2, 0-IPEA	cc-pVDZ	0.0015	0.2963	-0.0953	-0.0004	0.2964	0.0015	0.2963	0.0015	-0.0953	-0.0004	0.2964	0.0015	0.2978	-0.0957	0.2979	0.2978	-0.0957	0.2979
CASPT2, 0-IPEA	cc-pVTZ	0.0031	0.2896	-0.0846	-0.0008	0.2896	0.0031	0.2896	0.0031	-0.0846	-0.0008	0.2896	0.0031	0.2927	-0.0854	0.2927	0.2927	-0.0854	0.2927
CASPT2, S-IPEA	6-31G(d,p)	0.0006	0.2988	-0.0989	-0.0002	0.2990	0.0006	0.2988	0.0006	-0.0989	-0.0002	0.2990	0.0006	0.2994	-0.0991	0.2996	0.2994	-0.0991	0.2996
CASPT2, S-IPEA	cc-pVDZ	0.0015	0.2959	-0.0951	-0.0004	0.2967	0.0015	0.2959	0.0015	-0.0951	-0.0004	0.2967	0.0015	0.2974	-0.0955	0.2982	0.2974	-0.0955	0.2982
CASPT2, S-IPEA	cc-pVTZ	0.0031	0.2894	-0.0844	-0.0008	0.2896	0.0031	0.2894	0.0031	-0.0844	-0.0008	0.2896	0.0031	0.2925	-0.0852	0.2927	0.2925	-0.0852	0.2927
CASSCF	6-31G(d,p)	0.0007	0.2987	-0.0985	-0.0002	0.2987	0.0007	0.2987	0.0007	-0.0985	-0.0002	0.2987	0.0007	0.2994	-0.0987	0.2994	0.2994	-0.0987	0.2994
CASSCF	cc-pVDZ	0.0015	0.2956	-0.0939	-0.0004	0.2956	0.0015	0.2956	0.0015	-0.0939	-0.0004	0.2956	0.0015	0.2971	-0.0943	0.2971	0.2971	-0.0943	0.2971
CASSCF	cc-pVTZ	0.0031	0.2893	-0.0840	-0.0008	0.2893	0.0031	0.2893	0.0031	-0.0840	-0.0008	0.2893	0.0031	0.2924	-0.0848	0.2924	0.2924	-0.0848	0.2924
CASSCF	cc-pVQZ	0.0038	0.2868	-0.0803	-0.0010	0.2868	0.0038	0.2868	0.0038	-0.0803	-0.0010	0.2868	0.0038	0.2906	-0.0813	0.2906	0.2906	-0.0813	0.2906
DFT(B3LYP)	6-31G(d,p)	0.0007	0.2478	0.0030	0.0000	0.2478	0.0007	0.2478	0.0007	0.0030	0.0000	0.2478	0.0007	0.2485	0.0030	0.2485	0.2485	0.0030	0.2485
DFT(B3LYP)	cc-pVDZ	0.0015	0.2453	0.0063	0.0000	0.2453	0.0015	0.2453	0.0015	0.0063	0.0000	0.2453	0.0015	0.2468	0.0063	0.2468	0.2468	0.0063	0.2468
DFT(B3LYP)	cc-pVTZ	0.0034	0.2405	0.0122	0.0000	0.2405	0.0034	0.2405	0.0034	0.0122	0.0000	0.2405	0.0034	0.2439	0.0122	0.2439	0.2439	0.0122	0.2439
DFT(B3LYP)	cc-pVQZ	0.0045	0.2387	0.0136	0.0000	0.2387	0.0045	0.2387	0.0045	0.0136	0.0000	0.2387	0.0045	0.2432	0.0136	0.2432	0.2432	0.0136	0.2432
DFT(M06-2X)	6-31G(d,p)	0.0005	0.2480	0.0030	0.0000	0.2480	0.0005	0.2480	0.0005	0.0030	0.0000	0.2480	0.0005	0.2485	0.0030	0.2485	0.2485	0.0030	0.2485
DFT(M06-2X)	cc-pVDZ	0.0013	0.2455	0.0065	0.0000	0.2455	0.0013	0.2455	0.0013	0.0065	0.0000	0.2455	0.0013	0.2468	0.0065	0.2468	0.2468	0.0065	0.2468
DFT(M06-2X)	cc-pVTZ	0.0028	0.2409	0.0127	0.0000	0.2409	0.0028	0.2409	0.0028	0.0127	0.0000	0.2409	0.0028	0.2436	0.0127	0.2436	0.2436	0.0127	0.2436
DFT(M06-2X)	cc-pVQZ	0.0033	0.2389	0.0155	0.0000	0.2389	0.0033	0.2389	0.0033	0.0155	0.0000	0.2389	0.0033	0.2423	0.0155	0.2423	0.2423	0.0155	0.2423
DFT(CAM-B3LYP)	6-31G(d,p)	0.0006	0.2478	0.0031	0.0000	0.2478	0.0006	0.2478	0.0006	0.0031	0.0000	0.2478	0.0006	0.2485	0.0031	0.2485	0.2485	0.0031	0.2485
DFT(CAM-B3LYP)	cc-pVDZ	0.0014	0.2454	0.0065	0.0000	0.2454	0.0014	0.2454	0.0014	0.0065	0.0000	0.2454	0.0014	0.2468	0.0065	0.2468	0.2468	0.0065	0.2468
DFT(CAM-B3LYP)	cc-pVTZ	0.0031	0.2406	0.0125	0.0000	0.2406	0.0031	0.2406	0.0031	0.0125	0.0000	0.2406	0.0031	0.2437	0.0125	0.2437	0.2437	0.0125	0.2437
DFT(CAM-B3LYP)	cc-pVQZ	0.0040	0.2389	0.0140	0.0000	0.2389	0.0040	0.2389	0.0040	0.0140	0.0000	0.2389	0.0040	0.2430	0.0141	0.2430	0.2430	0.0141	0.2430

Table S23. Mulliken atomic spin densities obtained with chosen computational approaches for quinoid isomer of benzene molecule in the first electronic triplet excided state. In the right part of the table atomic spin densities of hydrogens are summed into heavy atoms they are connected to. Please note, that data for CASPT2 method are computed for equilibrium geometries obtained at this level of theory, however spin densities are computed basing on CASSCF wavefunction.

BZ(Q) R^{0}_{T1}

Method	Basis set	H1	C2	C3	H4	C5	H6	C7	H8	C9	H10	C11	H12	H1+C2	C3+H4	C5+H6	C7+H8	C9+J10	C11+H12
CASPT2, 0-IPEA	6-31G(d,p)	0.0027	0.7457	0.1253	0.0005	0.1253	0.0005	0.7457	0.0027	0.1253	0.0005	0.1253	0.0005	0.7484	0.1258	0.1258	0.7484	0.1258	0.1258
CASPT2, 0-IPEA	cc-pVDZ	0.0054	0.7439	0.1244	0.0010	0.1244	0.0010	0.7439	0.0054	0.1244	0.0010	0.1244	0.0010	0.7493	0.1254	0.1254	0.7493	0.1254	0.1254
CASPT2, 0-IPEA	cc-pVTZ	0.0112	0.7494	0.1178	0.0019	0.1178	0.0019	0.7494	0.0112	0.1178	0.0019	0.1178	0.0019	0.7606	0.1197	0.1197	0.7606	0.1197	0.1197
CASPT2, S-IPEA	6-31G(d,p)	0.0028	0.7654	0.1155	0.0004	0.1155	0.0004	0.7654	0.0028	0.1155	0.0004	0.1155	0.0004	0.7682	0.1159	0.1159	0.7682	0.1159	0.1159
CASPT2, S-IPEA	cc-pVDZ	0.0055	0.7639	0.1144	0.0009	0.1144	0.0009	0.7639	0.0055	0.1144	0.0009	0.1144	0.0009	0.7694	0.1153	0.1153	0.7694	0.1153	0.1153
CASPT2, S-IPEA	cc-pVTZ	0.0114	0.7638	0.1106	0.0018	0.1106	0.0018	0.7638	0.0114	0.1106	0.0018	0.1106	0.0018	0.7752	0.1124	0.1124	0.7752	0.1124	0.1124
CASSCF	6-31G(d,p)	0.0029	0.7751	0.1106	0.0004	0.1106	0.0004	0.7751	0.0029	0.1106	0.0004	0.1106	0.0004	0.7780	0.1110	0.1110	0.7780	0.1110	0.1110
CASSCF	cc-pVDZ	0.0058	0.7732	0.1096	0.0009	0.1096	0.0009	0.7732	0.0058	0.1096	0.0009	0.1096	0.0009	0.7790	0.1105	0.1105	0.7790	0.1105	0.1105
CASSCF	cc-pVTZ	0.0117	0.7718	0.1065	0.0018	0.1065	0.0018	0.7718	0.0117	0.1065	0.0018	0.1065	0.0018	0.7835	0.1083	0.1083	0.7835	0.1083	0.1083
CASSCF	cc-pVQZ	0.0150	0.7697	0.1053	0.0023	0.1053	0.0023	0.7697	0.0150	0.1053	0.0023	0.1053	0.0023	0.7847	0.1076	0.1076	0.7847	0.1076	0.1076
DFT(B3LYP)	6-31G(d,p)	-0.0430	0.8976	0.0805	-0.0078	0.0805	-0.0078	0.8976	-0.0430	0.0805	-0.0078	0.0805	-0.0078	0.8546	0.0727	0.0727	0.8546	0.0727	0.0727
DFT(B3LYP)	cc-pVDZ	-0.0382	0.8662	0.0931	-0.0071	0.0931	-0.0071	0.8662	-0.0382	0.0931	-0.0071	0.0931	-0.0071	0.8280	0.0860	0.0860	0.8280	0.0860	0.0860
DFT(B3LYP)	cc-pVTZ	-0.0318	0.8397	0.1029	-0.0068	0.1029	-0.0068	0.8397	-0.0318	0.1029	-0.0068	0.1029	-0.0068	0.8079	0.0960	0.0960	0.8079	0.0960	0.0960
DFT(B3LYP)	cc-pVQZ	-0.0360	0.8508	0.1017	-0.0091	0.1017	-0.0091	0.8508	-0.0360	0.1017	-0.0091	0.1017	-0.0091	0.8148	0.0926	0.0926	0.8148	0.0926	0.0926
DFT(M06-2X)	6-31G(d,p)	-0.0482	0.9436	0.0610	-0.0087	0.0610	-0.0087	0.9436	-0.0482	0.0610	-0.0087	0.0610	-0.0087	0.8954	0.0523	0.0523	0.8954	0.0523	0.0523
DFT(M06-2X)	cc-pVDZ	-0.0399	0.9061	0.0743	-0.0074	0.0743	-0.0074	0.9061	-0.0399	0.0743	-0.0074	0.0743	-0.0074	0.8662	0.0669	0.0669	0.8662	0.0669	0.0669
DFT(M06-2X)	cc-pVTZ	-0.0443	0.9097	0.0754	-0.0081	0.0754	-0.0081	0.9097	-0.0443	0.0754	-0.0081	0.0754	-0.0081	0.8655	0.0673	0.0673	0.8655	0.0673	0.0673
DFT(M06-2X)	cc-pVQZ	0.0074	0.8071	0.0800	0.0127	0.0800	0.0127	0.8071	0.0074	0.0800	0.0127	0.0800	0.0127	0.8145	0.0927	0.0927	0.8145	0.0927	0.0927
DFT(CAM-B3LYP)	6-31G(d,p)	-0.0441	0.9274	0.0657	-0.0073	0.0657	-0.0073	0.9274	-0.0441	0.0657	-0.0073	0.0657	-0.0073	0.8833	0.0584	0.0584	0.8833	0.0584	0.0584
DFT(CAM-B3LYP)	cc-pVDZ	-0.0388	0.8940	0.0789	-0.0066	0.0789	-0.0066	0.8940	-0.0388	0.0789	-0.0066	0.0789	-0.0066	0.8552	0.0724	0.0724	0.8552	0.0724	0.0724
DFT(CAM-B3LYP)	cc-pVTZ	-0.0346	0.8692	0.0890	-0.0063	0.0890	-0.0063	0.8692	-0.0346	0.0890	-0.0063	0.0890	-0.0063	0.8346	0.0827	0.0827	0.8346	0.0827	0.0827
DFT(CAM-B3LYP)	cc-pVQZ	-0.0423	0.8814	0.0884	-0.0080	0.0884	-0.0080	0.8814	-0.0423	0.0884	-0.0080	0.0884	-0.0080	0.8391	0.0804	0.0804	0.8391	0.0804	0.0804

Table S24. Mulliken atomic spin densities obtained with chosen computational approaches for antiquinoid isomer of benzene molecule in the first electronic triplet excided state. In the right part of the table atomic spin densities of hydrogens are summed into heavy atoms they are connected to. Please note, that data for CASPT2 method are computed for equilibrium geometries obtained at this level of theory, however spin densities are computed basing on CASSCF wavefunction.

BZ(AQ) R^{0}_{T1}

Method	Basis set	H1	C2	C3	H4	C5	H6	C7	H8	C9	H10	C11	H12	H1+C2	C3+H4	C5+H6	C7+H8	C9+J10	C11+H12
CASPT2, 0-IPEA	6-31G(d,p)	0.0019	0.5260	-0.0556	-0.0001	0.5260	0.0019	0.5260	0.0019	-0.0556	-0.0001	0.5260	0.0019	0.5279	-0.0557	0.5279	0.5279	-0.0557	0.5279
CASPT2, 0-IPEA	cc-pVDZ	0.0037	0.5249	-0.0570	-0.0002	0.5249	0.0037	0.5249	0.0037	-0.0570	-0.0002	0.5249	0.0037	0.5286	-0.0572	0.5286	0.5286	-0.0572	0.5286
CASPT2, 0-IPEA	cc-pVTZ	0.0077	0.5275	-0.0699	-0.0007	0.5275	0.0077	0.5275	0.0077	-0.0699	-0.0007	0.5275	0.0077	0.5352	-0.0706	0.5352	0.5352	-0.0706	0.5352
CASPT2, S-IPEA	6-31G(d,p)	0.0019	0.5377	-0.0789	-0.0002	0.5377	0.0019	0.5377	0.0019	-0.0789	-0.0002	0.5377	0.0019	0.5396	-0.0791	0.5396	0.5396	-0.0791	0.5396
CASPT2, S-IPEA	cc-pVDZ	0.0038	0.5363	-0.0798	-0.0004	0.5363	0.0038	0.5363	0.0038	-0.0798	-0.0004	0.5363	0.0038	0.5401	-0.0802	0.5401	0.5401	-0.0802	0.5401
CASPT2, S-IPEA	cc-pVTZ	0.0078	0.5341	-0.0830	-0.0008	0.5341	0.0078	0.5341	0.0078	-0.0830	-0.0008	0.5341	0.0078	0.5419	-0.0838	0.5419	0.5419	-0.0838	0.5419
CASSCF	6-31G(d,p)	0.0019	0.5383	-0.0803	-0.0002	0.5383	0.0019	0.5383	0.0019	-0.0803	-0.0002	0.5383	0.0019	0.5402	-0.0805	0.5402	0.5402	-0.0805	0.5402
CASSCF	cc-pVDZ	0.0040	0.5370	-0.0815	-0.0004	0.5370	0.0040	0.5370	0.0040	-0.0815	-0.0004	0.5370	0.0040	0.5410	-0.0819	0.5410	0.5410	-0.0819	0.5410
CASSCF	cc-pVTZ	0.0079	0.5341	-0.0832	-0.0009	0.5341	0.0079	0.5341	0.0079	-0.0832	-0.0009	0.5341	0.0079	0.5420	-0.0841	0.5420	0.5420	-0.0841	0.5420
CASSCF	cc-pVQZ	0.0102	0.5316	-0.0826	-0.0009	0.5316	0.0102	0.5316	0.0102	-0.0826	-0.0009	0.5316	0.0102	0.5418	-0.0835	0.5418	0.5418	-0.0835	0.5418
DFT(B3LYP)	6-31G(d,p)	-0.0330	0.6613	-0.2640	0.0073	0.6613	-0.0330	0.6613	-0.0330	-0.2640	0.0073	0.6613	-0.0330	0.6283	-0.2567	0.6283	0.6283	-0.2567	0.6283
DFT(B3LYP)	cc-pVDZ	-0.0295	0.6392	-0.2258	0.0064	0.6392	-0.0295	0.6392	-0.0295	-0.2258	0.0064	0.6392	-0.0295	0.6097	-0.2194	0.6097	0.6097	-0.2194	0.6097
DFT(B3LYP)	cc-pVTZ	-0.0252	0.6175	-0.1892	0.0046	0.6175	-0.0252	0.6175	-0.0252	-0.1892	0.0046	0.6175	-0.0252	0.5923	-0.1847	0.5923	0.5923	-0.1847	0.5923
DFT(B3LYP)	cc-pVQZ	-0.0288	0.6258	-0.1972	0.0032	0.6258	-0.0288	0.6258	-0.0288	-0.1972	0.0032	0.6258	-0.0288	0.5970	-0.1940	0.5970	0.5970	-0.1940	0.5970
DFT(M06-2X)	6-31G(d,p)	-0.0353	0.6680	-0.2712	0.0059	0.6680	-0.0353	0.6680	-0.0353	-0.2712	0.0059	0.6680	-0.0353	0.6327	-0.2654	0.6327	0.6327	-0.2654	0.6327
DFT(M06-2X)	cc-pVDZ	-0.0292	0.6531	-0.2522	0.0045	0.6531	-0.0292	0.6531	-0.0292	-0.2522	0.0045	0.6531	-0.0292	0.6239	-0.2477	0.6239	0.6239	-0.2477	0.6239
DFT(M06-2X)	cc-pVTZ	-0.0385	0.6796	-0.2982	0.0160	0.6796	-0.0385	0.6796	-0.0385	-0.2982	0.0160	0.6796	-0.0385	0.6411	-0.2822	0.6411	0.6411	-0.2822	0.6411
DFT(M06-2X)	cc-pVQZ	0.0036	0.6017	-0.2264	0.0158	0.6017	0.0036	0.6017	0.0036	-0.2264	0.0158	0.6017	0.0036	0.6053	-0.2107	0.6053	0.6053	-0.2107	0.6053
DFT(CAM-B3LYP)	6-31G(d,p)	-0.0340	0.6870	-0.3152	0.0091	0.6870	-0.0340	0.6870	-0.0340	-0.3152	0.0091	0.6870	-0.0340	0.6530	-0.3061	0.6530	0.6530	-0.3061	0.6530
DFT(CAM-B3LYP)	cc-pVDZ	-0.0300	0.6627	-0.2733	0.0080	0.6627	-0.0300	0.6627	-0.0300	-0.2733	0.0080	0.6627	-0.0300	0.6327	-0.2653	0.6327	0.6327	-0.2653	0.6327
DFT(CAM-B3LYP)	cc-pVTZ	-0.0271	0.6408	-0.2342	0.0067	0.6408	-0.0271	0.6408	-0.0271	-0.2342	0.0067	0.6408	-0.0271	0.6137	-0.2274	0.6137	0.6137	-0.2274	0.6137
DFT(CAM-B3LYP)	cc-pVQZ	-0.0328	0.6487	-0.2387	0.0070	0.6487	-0.0328	0.6487	-0.0328	-0.2387	0.0070	0.6487	-0.0328	0.6159	-0.2317	0.6159	0.6159	-0.2317	0.6159

Table S25. Mulliken atomic spin densities for benzene molecule in the ground electronic state predicted as combination (**R^{-} + R^{+} - R^{0}_{T1}**) of atomic spin densities computed for other electronic states structures in their quinoid variants. In the right part of the table there are data for summed spin densities of heavy atoms and hydrogens connected to them. Please note, that data for CASPT2 method are computed for equilibrium geometries obtained at this level of theory, however spin densities are computed basing on CASSCF wavefunction.

BZ(Q) $R^{-} + R^{+} - R^{0}_{T1}$

Method	Basis set	H1	C2	C3	H4	C5	H6	C7	H8	C9	H10	C11	H12	H1+C2	C3+H4	C5+H6	C7+H8	C9+J10	C11+H12
**CASPT2, 0-IPEA**	**6-31G(d,p)**	0.0003	0.1153	-0.0583	-0.0001	-0.0571	-0.0001	0.1153	0.0003	-0.0583	-0.0001	-0.0571	-0.0001	0.1156	-0.0584	-0.0572	0.1156	-0.0584	-0.0572
**CASPT2, 0-IPEA**	**cc-pVDZ**	0.0007	0.1055	-0.0529	-0.0003	-0.0529	-0.0003	0.1055	0.0007	-0.0529	-0.0003	-0.0529	-0.0003	0.1062	-0.0532	-0.0532	0.1062	-0.0532	-0.0532
**CASPT2, 0-IPEA**	**cc-pVTZ**	0.0021	0.0735	-0.0376	-0.0003	-0.0376	-0.0003	0.0735	0.0021	-0.0376	-0.0003	-0.0376	-0.0003	0.0756	-0.0379	-0.0379	0.0756	-0.0379	-0.0379
**CASPT2, S-IPEA**	**6-31G(d,p)**	0.0002	0.0956	-0.0479	0.0000	-0.0477	0.0000	0.0956	0.0002	-0.0479	0.0000	-0.0477	0.0000	0.0958	-0.0479	-0.0477	0.0958	-0.0479	-0.0477
**CASPT2, S-IPEA**	**cc-pVDZ**	0.0006	0.0854	-0.0428	-0.0002	-0.0428	-0.0002	0.0854	0.0006	-0.0428	-0.0002	-0.0428	-0.0002	0.0860	-0.0430	-0.0430	0.0860	-0.0430	-0.0430
**CASPT2, S-IPEA**	**cc-pVTZ**	0.0019	0.0590	-0.0303	-0.0002	-0.0303	-0.0002	0.0590	0.0019	-0.0303	-0.0002	-0.0303	-0.0002	0.0609	-0.0305	-0.0305	0.0609	-0.0305	-0.0305
**CASSCF**	**6-31G(d,p)**	0.0002	0.0858	-0.0430	0.0000	-0.0430	0.0000	0.0858	0.0002	-0.0430	0.0000	-0.0430	0.0000	0.0860	-0.0430	-0.0430	0.0860	-0.0430	-0.0430
**CASSCF**	**cc-pVDZ**	0.0005	0.0742	-0.0372	-0.0002	-0.0372	-0.0002	0.0742	0.0005	-0.0372	-0.0002	-0.0372	-0.0002	0.0747	-0.0374	-0.0374	0.0747	-0.0374	-0.0374
**CASSCF**	**cc-pVTZ**	0.0018	0.0506	-0.0261	-0.0002	-0.0261	-0.0002	0.0506	0.0018	-0.0261	-0.0002	-0.0261	-0.0002	0.0524	-0.0263	-0.0263	0.0524	-0.0263	-0.0263
**CASSCF**	**cc-pVQZ**	0.0062	0.0438	-0.0256	0.0007	-0.0256	0.0007	0.0438	0.0062	-0.0256	0.0007	-0.0256	0.0007	0.0500	-0.0249	-0.0249	0.0500	-0.0249	-0.0249
**DFT(B3LYP)**	**6-31G(d,p)**	0.0461	-0.1780	0.0575	0.0084	0.0575	0.0084	-0.1780	0.0461	0.0575	0.0084	0.0575	0.0084	-0.1319	0.0660	0.0660	-0.1319	0.0660	0.0660
**DFT(B3LYP)**	**cc-pVDZ**	0.0445	-0.1580	0.0483	0.0084	0.0483	0.0084	-0.1580	0.0445	0.0483	0.0084	0.0483	0.0084	-0.1135	0.0567	0.0567	-0.1135	0.0567	0.0567
**DFT(B3LYP)**	**cc-pVTZ**	0.0463	-0.1517	0.0428	0.0099	0.0428	0.0099	-0.1517	0.0463	0.0428	0.0099	0.0428	0.0099	-0.1053	0.0527	0.0527	-0.1053	0.0527	0.0527
**DFT(B3LYP)**	**cc-pVQZ**	0.0612	-0.1710	0.0404	0.0145	0.0404	0.0145	-0.1710	0.0612	0.0404	0.0145	0.0404	0.0145	-0.1098	0.0549	0.0549	-0.1098	0.0549	0.0549
**DFT(M06-2X)**	**6-31G(d,p)**	0.0509	-0.2292	0.0799	0.0093	0.0799	0.0093	-0.2292	0.0509	0.0799	0.0093	0.0799	0.0093	-0.1783	0.0892	0.0892	-0.1783	0.0892	0.0892
**DFT(M06-2X)**	**cc-pVDZ**	0.0454	-0.2033	0.0704	0.0085	0.0704	0.0085	-0.2033	0.0454	0.0704	0.0085	0.0704	0.0085	-0.1578	0.0789	0.0789	-0.1578	0.0789	0.0789
**DFT(M06-2X)**	**cc-pVTZ**	0.0566	-0.2261	0.0740	0.0107	0.0740	0.0107	-0.2261	0.0566	0.0740	0.0107	0.0740	0.0107	-0.1694	0.0847	0.0847	-0.1694	0.0847	0.0847
**DFT(M06-2X)**	**cc-pVQZ**	0.0113	-0.1317	0.0687	-0.0085	0.0687	-0.0085	-0.1317	0.0113	0.0687	-0.0085	0.0687	-0.0085	-0.1204	0.0602	0.0602	-0.1204	0.0602	0.0602
**DFT(CAM-B3LYP)**	**6-31G(d,p)**	0.0471	-0.2091	0.0731	0.0079	0.0731	0.0079	-0.2091	0.0471	0.0731	0.0079	0.0731	0.0079	-0.1620	0.0810	0.0810	-0.1620	0.0810	0.0810
**DFT(CAM-B3LYP)**	**cc-pVDZ**	0.0148	-0.0515	0.0148	0.0036	0.0148	0.0036	-0.0515	0.0148	0.0148	0.0036	0.0148	0.0036	-0.0368	0.0184	0.0184	-0.0368	0.0184	0.0184
**DFT(CAM-B3LYP)**	**cc-pVTZ**	0.0482	-0.1826	0.0581	0.0091	0.0581	0.0091	-0.1826	0.0482	0.0581	0.0091	0.0581	0.0091	-0.1344	0.0672	0.0672	-0.1344	0.0672	0.0672
**DFT(CAM-B3LYP)**	**cc-pVQZ**	0.0655	-0.2024	0.0555	0.0129	0.0555	0.0129	-0.2024	0.0655	0.0555	0.0129	0.0555	0.0129	-0.1369	0.0684	0.0684	-0.1369	0.0684	0.0684

Table S26. Mulliken atomic spin densities for benzene molecule in the ground electronic state predicted as combination (**$R^- + R^+ - R^0_{T1}$**) of atomic spin densities computed for other electronic states structures in their antiquinoid variants. In the right part of the table there are data for summed spin densities of heavy atoms and hydrogens connected to them. Please note, that data for CASPT2 method are computed for equilibrium geometries obtained at this level of theory, however spin densities are computed basing on CASSCF wavefunction. TBD – to be determined.

BZ(AQ) $R^- + R^+ - R^0_{T1}$

Method	Basis set	H1	C2	C3	H4	C5	H6	C7	H8	C9	H10	C11	H12	H1+C2	C3+H4	C5+H6	C7+H8	C9+J10	C11+H12
CASPT2, 0-IPEA	6-31G(d,p)	0.0001	0.0657	-0.1322	-0.0003	0.0664	0.0001	0.0657	0.0001	-0.1322	-0.0003	0.0664	0.0001	0.0658	-0.1325	0.0665	0.0658	-0.1325	0.0665
CASPT2, 0-IPEA	cc-pVDZ	0.0005	0.0608	-0.1221	-0.0006	0.0609	0.0005	0.0608	0.0005	-0.1221	-0.0006	0.0609	0.0005	0.0613	-0.1227	0.0614	0.0613	-0.1227	0.0614
CASPT2, 0-IPEA	cc-pVTZ	0.0012	0.0463	-0.0879	-0.0009	0.0436	0.0013	0.0463	0.0013	-0.0877	-0.0009	0.0463	0.0013	0.0448	-0.0888	0.0449	0.0539	-0.0886	0.0439
CASPT2, S-IPEA	6-31G(d,p)	0.0001	0.0541	-0.1086	-0.0002	0.0543	0.0001	0.0541	0.0001	-0.1086	-0.0002	0.0543	0.0001	0.0542	-0.1088	0.0544	0.0542	-0.1088	0.0544
CASPT2, S-IPEA	cc-pVDZ	0.0004	0.0489	-0.0989	-0.0004	0.0497	0.0004	0.0489	0.0004	-0.0989	-0.0004	0.0497	0.0004	0.0493	-0.0993	0.0501	0.0493	-0.0993	0.0501
CASPT2, S-IPEA	cc-pVTZ	0.0012	0.0365	-0.0749	-0.0008	0.0367	0.0012	0.0365	0.0012	-0.0749	-0.0008	0.0367	0.0012	0.0377	-0.0757	0.0379	0.0377	-0.0757	0.0379
CASSCF	6-31G(d,p)	0.0002	0.0532	-0.1065	-0.0002	0.0532	0.0002	0.0532	0.0002	-0.1065	-0.0002	0.0532	0.0002	0.0534	-0.1067	0.0534	0.0534	-0.1067	0.0534
CASSCF	cc-pVDZ	0.0003	0.0471	-0.0945	-0.0004	0.0471	0.0003	0.0471	0.0003	-0.0945	-0.0004	0.0471	0.0003	0.0474	-0.0949	0.0474	0.0474	-0.0949	0.0474
CASSCF	cc-pVTZ	0.0012	0.0356	-0.0730	-0.0007	0.0356	0.0012	0.0356	0.0012	-0.0730	-0.0007	0.0356	0.0012	0.0368	-0.0737	0.0368	0.0368	-0.0737	0.0368
CASSCF	cc-pVQZ	0.0040	0.0321	-0.0715	-0.0008	0.0321	0.0040	0.0321	0.0040	-0.0715	-0.0008	0.0321	0.0040	0.0361	-0.0723	0.0361	0.0361	-0.0723	0.0361
DFT(B3LYP)	6-31G(d,p)	0.0352	-0.1681	0.2730	-0.0073	-0.1681	0.0352	-0.1681	0.0352	0.2730	-0.0073	-0.1681	0.0352	-0.1329	0.2658	-0.1329	-0.1329	0.2658	-0.1329
DFT(B3LYP)	cc-pVDZ	0.0339	-0.1520	0.2426	-0.0064	-0.1520	0.0339	-0.1520	0.0339	0.2426	-0.0064	-0.1520	0.0339	-0.1181	0.2362	-0.1181	-0.1181	0.2362	-0.1181
DFT(B3LYP)	cc-pVTZ	0.0355	-0.1411	0.2157	-0.0045	-0.1411	0.0355	-0.1411	0.0355	0.2157	-0.0045	-0.1411	0.0355	-0.1056	0.2112	-0.1056	-0.1056	0.2112	-0.1056
DFT(B3LYP)	cc-pVQZ	0.0467	-0.1540	0.2175	-0.0030	-0.1540	0.0467	-0.1540	0.0467	0.2175	-0.0030	-0.1540	0.0467	-0.1073	0.2145	-0.1073	-0.1073	0.2145	-0.1073
DFT(M06-2X)	6-31G(d,p)	0.0372	-0.1746	0.2808	-0.0059	-0.1746	0.0372	-0.1746	0.0372	0.2808	-0.0059	-0.1746	0.0372	-0.1375	0.2749	-0.1375	-0.1375	0.2749	-0.1375
DFT(M06-2X)	cc-pVDZ	0.0331	-0.1660	0.2702	-0.0045	-0.1660	0.0331	-0.1660	0.0331	0.2702	-0.0045	-0.1660	0.0331	-0.1329	0.2657	-0.1329	-0.1329	0.2657	-0.1329
DFT(M06-2X)	cc-pVTZ	0.0473	-0.2031	0.3275	-0.0160	-0.2031	0.0473	-0.2031	0.0473	0.3275	-0.0160	-0.2031	0.0473	-0.1558	0.3115	-0.1558	-0.1558	0.3115	-0.1558
DFT(M06-2X)	cc-pVQZ	0.0099	-0.1302	0.2562	-0.0156	-0.1302	0.0099	-0.1302	0.0099	0.2562	-0.0156	-0.1302	0.0099	-0.1203	0.2406	-0.1203	-0.1203	0.2406	-0.1203
DFT(CAM-B3LYP)	6-31G(d,p)	0.0361	-0.1939	0.3248	-0.0091	-0.1939	0.0361	-0.1939	0.0361	0.3248	-0.0091	-0.1939	0.0361	-0.1579	0.3157	-0.1579	-0.1579	0.3157	-0.1579
DFT(CAM-B3LYP)	cc-pVDZ	0.0129	-0.0869	0.1517	-0.0035	-0.0869	0.0129	-0.0869	0.0129	0.1517	-0.0035	-0.0869	0.0129	-0.0741	0.1482	-0.0741	-0.0741	0.1482	-0.0741
DFT(CAM-B3LYP)	cc-pVTZ	0.0368	-0.1648	0.2627	-0.0067	-0.1648	0.0368	-0.1648	0.0368	0.2627	-0.0067	-0.1648	0.0368	-0.1280	0.2561	-0.1280	-0.1280	0.2561	-0.1280
DFT(CAM-B3LYP)	cc-pVQZ	0.0493	-0.1773	0.2627	-0.0068	-0.1773	0.0493	-0.1773	0.0493	0.2627	-0.0068	-0.1773	0.0493	-0.1280	0.2559	-0.1280	-0.1280	0.2559	-0.1280

Table S27. Mulliken atomic spin densities obtained with chosen computational approaches for anionic form of cyclobutadiene. In the right part of the table atomic spin densities of hydrogens are summed into heavy atoms they are connected to. Please note, that data for CASPT2 method are computed for equilibrium geometries obtained at this level of theory, however spin densities are computed basing on CASSCF wavefunction.

CBDE R^{-}

Method	Basis set	C1	H2	C3	H4	C5	H6	C7	H8	C1+H2	C3+H4	C5+H6	C7+H8
CASPT2, 0-IPEA	6-31G(d,p)	0.2511	0.0011	0.2468	0.0011	0.2511	0.0011	0.2468	0.0011	0.2522	0.2479	0.2522	0.2479
CASPT2, 0-IPEA	cc-pVDZ	0.2493	0.0022	0.2465	0.0021	0.2492	0.0022	0.2465	0.0021	0.2515	0.2486	0.2514	0.2486
CASPT2, 0-IPEA	cc-pVTZ	0.2440	0.0049	0.2462	0.0049	0.2440	0.0049	0.2462	0.0049	0.2489	0.2511	0.2489	0.2511
CASPT2, S-IPEA	6-31G(d,p)	0.2490	0.0011	0.2488	0.0011	0.2490	0.0011	0.2488	0.0011	0.2501	0.2499	0.2501	0.2499
CASPT2, S-IPEA	cc-pVDZ	0.2484	0.0022	0.2473	0.0022	0.2484	0.0022	0.2473	0.0022	0.2506	0.2495	0.2506	0.2495
CASPT2, S-IPEA	cc-pVTZ	0.2455	0.0049	0.2447	0.0049	0.2453	0.0049	0.2448	0.0049	0.2504	0.2496	0.2502	0.2497
CASSCF	6-31G(d,p)	0.2489	0.0011	0.2489	0.0011	0.2489	0.0011	0.2489	0.0011	0.2500	0.2500	0.2500	0.2500
CASSCF	cc-pVDZ	0.2478	0.0022	0.2478	0.0022	0.2478	0.0022	0.2478	0.0022	0.2500	0.2500	0.2500	0.2500
CASSCF	cc-pVTZ	0.2448	0.0050	0.2452	0.0050	0.2448	0.0050	0.2452	0.0050	0.2498	0.2502	0.2498	0.2502
CASSCF	cc-pVQZ	0.2402	0.0097	0.2405	0.0097	0.2402	0.0097	0.2405	0.0097	0.2499	0.2502	0.2499	0.2502
DFT(B3LYP)	6-31G(d,p)	0.2652	-0.0152	0.2652	-0.0152	0.2652	-0.0152	0.2652	-0.0152	0.2500	0.2500	0.2500	0.2500
DFT(B3LYP)	cc-pVDZ	0.2644	-0.0144	0.2644	-0.0144	0.2644	-0.0144	0.2644	-0.0144	0.2500	0.2500	0.2500	0.2500
DFT(B3LYP)	cc-pVTZ	0.2613	-0.0112	0.2613	-0.0112	0.2612	-0.0112	0.2612	-0.0112	0.2500	0.2500	0.2500	0.2500
DFT(B3LYP)	cc-pVQZ	0.2605	-0.0105	0.2605	-0.0105	0.2605	-0.0105	0.2605	-0.0105	0.2500	0.2500	0.2500	0.2500
DFT(M06-2X)	6-31G(d,p)	0.2666	-0.0167	0.2667	-0.0167	0.2667	-0.0167	0.2667	-0.0167	0.2500	0.2500	0.2500	0.2500
DFT(M06-2X)	cc-pVDZ	0.2642	-0.0142	0.2642	-0.0142	0.2642	-0.0142	0.2642	-0.0142	0.2500	0.2500	0.2500	0.2500
DFT(M06-2X)	cc-pVTZ	0.2791	-0.0291	0.2791	-0.0291	0.2791	-0.0291	0.2791	-0.0291	0.2500	0.2500	0.2500	0.2500
DFT(M06-2X)	cc-pVQZ	0.2315	0.0185	0.2315	0.0185	0.2315	0.0185	0.2315	0.0185	0.2500	0.2500	0.2500	0.2500
DFT(CAM-B3LYP)	6-31G(d,p)	0.2652	-0.0152	0.2652	-0.0152	0.2652	-0.0152	0.2652	-0.0152	0.2500	0.2500	0.2500	0.2500
DFT(CAM-B3LYP)	cc-pVDZ	0.2643	-0.0143	0.2643	-0.0143	0.2643	-0.0143	0.2643	-0.0143	0.2500	0.2500	0.2500	0.2500
DFT(CAM-B3LYP)	cc-pVTZ	0.2622	-0.0122	0.2622	-0.0122	0.2622	-0.0122	0.2621	-0.0122	0.2500	0.2500	0.2500	0.2500
DFT(CAM-B3LYP)	cc-pVQZ	0.2791	-0.0291	0.2791	-0.0291	0.2791	-0.0291	0.2791	-0.0291	0.2500	0.2500	0.2500	0.2500

Table S28. Mulliken atomic spin densities obtained with chosen computational approaches for cationic form of cyclobutadiene. In the right part of the table atomic spin densities of hydrogens are summed into heavy atoms they are connected to. Please note, that data for CASPT2 method are computed for equilibrium geometries obtained at this level of theory, however spin densities are computed basing on CASSCF wavefunction.

CBDE R$^+$

Method	Basis set	C1	H2	C3	H4	C5	H6	C7	H8	C1+H2	C3+H4	C5+H6	C7+H8
CASPT2, 0-IPEA	6-31G(d,p)	0.2489	0.0007	0.2497	0.0007	0.2489	0.0007	0.2497	0.0007	0.2496	0.2504	0.2496	0.2504
CASPT2, 0-IPEA	cc-pVDZ	0.2484	0.0015	0.2485	0.0015	0.2484	0.0015	0.2485	0.0015	0.2499	0.2500	0.2499	0.2500
CASPT2, 0-IPEA	cc-pVTZ	0.2474	0.0028	0.2470	0.0028	0.2474	0.0028	0.2470	0.0028	0.2502	0.2498	0.2502	0.2498
CASPT2, S-IPEA	6-31G(d,p)	0.2490	0.0007	0.2496	0.0007	0.2490	0.0007	0.2496	0.0007	0.2497	0.2503	0.2497	0.2503
CASPT2, S-IPEA	cc-pVDZ	0.2485	0.0015	0.2485	0.0015	0.2485	0.0015	0.2484	0.0015	0.2500	0.2500	0.2500	0.2499
CASPT2, S-IPEA	cc-pVTZ	0.2472	0.0028	0.2472	0.0028	0.2472	0.0028	0.2472	0.0028	0.2500	0.2500	0.2500	0.2500
CASSCF	6-31G(d,p)	0.2493	0.0008	0.2492	0.0008	0.2493	0.0008	0.2492	0.0008	0.2501	0.2500	0.2501	0.2500
CASSCF	cc-pVDZ	0.2484	0.0016	0.2484	0.0016	0.2484	0.0016	0.2484	0.0016	0.2500	0.2500	0.2500	0.2500
CASSCF	cc-pVTZ	0.2472	0.0029	0.2470	0.0029	0.2472	0.0029	0.2470	0.0029	0.2501	0.2499	0.2501	0.2499
CASSCF	cc-pVQZ	0.2466	0.0034	0.2465	0.0034	0.2466	0.0034	0.2465	0.0034	0.2500	0.2499	0.2500	0.2499
DFT(B3LYP)	6-31G(d,p)	0.2620	-0.0120	0.2620	-0.0120	0.2620	-0.0120	0.2620	-0.0120	0.2500	0.2500	0.2500	0.2500
DFT(B3LYP)	cc-pVDZ	0.2622	-0.0122	0.2622	-0.0122	0.2622	-0.0122	0.2622	-0.0122	0.2500	0.2500	0.2500	0.2500
DFT(B3LYP)	cc-pVTZ	0.2568	-0.0068	0.2568	-0.0068	0.2568	-0.0068	0.2568	-0.0068	0.2500	0.2500	0.2500	0.2500
DFT(B3LYP)	cc-pVQZ	0.2584	-0.0084	0.2585	-0.0084	0.2584	-0.0084	0.2585	-0.0084	0.2500	0.2500	0.2500	0.2500
DFT(M06-2X)	6-31G(d,p)	0.2607	-0.0107	0.2607	-0.0107	0.2607	-0.0107	0.2607	-0.0107	0.2500	0.2500	0.2500	0.2500
DFT(M06-2X)	cc-pVDZ	0.2606	-0.0106	0.2606	-0.0106	0.2606	-0.0106	0.2606	-0.0106	0.2500	0.2500	0.2500	0.2500
DFT(M06-2X)	cc-pVTZ	0.2589	-0.0089	0.2589	-0.0089	0.2589	-0.0089	0.2589	-0.0089	0.2500	0.2500	0.2500	0.2500
DFT(M06-2X)	cc-pVQZ	0.2468	0.0032	0.2468	0.0032	0.2468	0.0032	0.2468	0.0032	0.2500	0.2500	0.2500	0.2500
DFT(CAM-B3LYP)	6-31G(d,p)	0.2618	-0.0118	0.2618	-0.0118	0.2618	-0.0118	0.2618	-0.0118	0.2500	0.2500	0.2500	0.2500
DFT(CAM-B3LYP)	cc-pVDZ	0.2620	-0.0120	0.2620	-0.0120	0.2619	-0.0120	0.2619	-0.0120	0.2500	0.2500	0.2500	0.2500
DFT(CAM-B3LYP)	cc-pVTZ	0.2571	-0.0071	0.2571	-0.0071	0.2571	-0.0071	0.2571	-0.0071	0.2500	0.2500	0.2500	0.2500
DFT(CAM-B3LYP)	cc-pVQZ	0.2595	-0.0095	0.2595	-0.0095	0.2595	-0.0095	0.2595	-0.0095	0.2500	0.2500	0.2500	0.2500

Table S29. Mulliken atomic spin densities obtained with chosen computational approaches for the first triplet excited state of cyclobutadiene. In the right part of the table atomic spin densities of hydrogens are summed into heavy atoms they are connected to. Please note, that data for CASPT2 method are computed for equilibrium geometries obtained at this level of theory, however spin densities are computed basing on CASSCF wavefunction.

CBDE R^0_{T1}

Method	Basis set	C1	H2	C3	H4	C5	H6	C7	H8	C1+H2	C3+H4	C5+H6	C7+H8
CASPT2, 0-IPEA	6-31G(d,p)	0.4981	0.0019	0.4981	0.0019	0.4982	0.0019	0.4981	0.0019	0.5000	0.5000	0.5001	0.5000
CASPT2, 0-IPEA	cc-pVDZ	0.4963	0.0037	0.4962	0.0037	0.4963	0.0037	0.4964	0.0037	0.5000	0.4999	0.5000	0.5001
CASPT2, 0-IPEA	cc-pVTZ	0.4926	0.0074	0.4926	0.0074	0.4926	0.0074	0.4926	0.0074	0.5000	0.5000	0.5000	0.5000
CASPT2, S-IPEA	6-31G(d,p)	0.4981	0.0019	0.4981	0.0019	0.4982	0.0019	0.4981	0.0019	0.5000	0.5000	0.5001	0.5000
CASPT2, S-IPEA	cc-pVDZ	0.4965	0.0037	0.4960	0.0037	0.4966	0.0037	0.4959	0.0037	0.5002	0.4997	0.5003	0.4996
CASPT2, S-IPEA	cc-pVTZ	0.4931	0.0073	0.4922	0.0073	0.4930	0.0074	0.4923	0.0074	0.5004	0.4995	0.5004	0.4997
CASSCF	6-31G(d,p)	0.4981	0.0019	0.4981	0.0019	0.4981	0.0019	0.4981	0.0019	0.5000	0.5000	0.5000	0.5000
CASSCF	cc-pVDZ	0.4961	0.0039	0.4961	0.0039	0.4961	0.0039	0.4961	0.0039	0.5000	0.5000	0.5000	0.5000
CASSCF	cc-pVTZ	0.4925	0.0075	0.4925	0.0075	0.4925	0.0075	0.4925	0.0075	0.5000	0.5000	0.5000	0.5000
CASSCF	cc-pVQZ	0.4896	0.0104	0.4896	0.0104	0.4896	0.0104	0.4896	0.0104	0.5000	0.5000	0.5000	0.5000
DFT(B3LYP)	6-31G(d,p)	0.5268	-0.0268	0.5268	-0.0268	0.5268	-0.0268	0.5268	-0.0268	0.5000	0.5000	0.5000	0.5000
DFT(B3LYP)	cc-pVDZ	0.5260	-0.0260	0.5260	-0.0260	0.5260	-0.0260	0.5260	-0.0260	0.5000	0.5000	0.5000	0.5000
DFT(B3LYP)	cc-pVTZ	0.5172	-0.0172	0.5172	-0.0172	0.5172	-0.0172	0.5172	-0.0172	0.5000	0.5000	0.5000	0.5000
DFT(B3LYP)	cc-pVQZ	0.5186	-0.0186	0.5186	-0.0186	0.5186	-0.0186	0.5186	-0.0186	0.5000	0.5000	0.5000	0.5000
DFT(M06-2X)	6-31G(d,p)	0.5279	-0.0279	0.5279	-0.0279	0.5279	-0.0279	0.5279	-0.0279	0.5000	0.5000	0.5000	0.5000
DFT(M06-2X)	cc-pVDZ	0.5246	-0.0246	0.5246	-0.0246	0.5246	-0.0246	0.5246	-0.0246	0.5000	0.5000	0.5000	0.5000
DFT(M06-2X)	cc-pVTZ	0.5330	-0.0330	0.5330	-0.0330	0.5330	-0.0330	0.5330	-0.0330	0.5000	0.5000	0.5000	0.5000
DFT(M06-2X)	cc-pVQZ	0.4744	0.0256	0.4744	0.0256	0.4744	0.0256	0.4744	0.0256	0.5000	0.5000	0.5000	0.5000
DFT(CAM-B3LYP)	6-31G(d,p)	0.5265	-0.0265	0.5265	-0.0265	0.5265	-0.0265	0.5265	-0.0265	0.5000	0.5000	0.5000	0.5000
DFT(CAM-B3LYP)	cc-pVDZ	0.5256	-0.0256	0.5256	-0.0256	0.5256	-0.0256	0.5256	-0.0256	0.5000	0.5000	0.5000	0.5000
DFT(CAM-B3LYP)	cc-pVTZ	0.5181	-0.0181	0.5181	-0.0181	0.5181	-0.0181	0.5181	-0.0181	0.5000	0.5000	0.5000	0.5000
DFT(CAM-B3LYP)	cc-pVQZ	0.5218	-0.0218	0.5218	-0.0218	0.5218	-0.0218	0.5218	-0.0218	0.5000	0.5000	0.5000	0.5000

Table S30. Mulliken atomic spin densities for cyclobutadiene in the ground electronic state predicted as combination ($\mathbf{R^{-} + R^{+} - R^{0}_{T1}}$) of atomic spin densities computed for other electronic states structures. In the right part of the table there are data for summed spin densities of heavy atoms and hydrogens connected to them. Please note, that data for CASPT2 method are computed for equilibrium geometries obtained at this level of theory, however spin densities are computed basing on CASSCF wavefunction.

CBDE $R^{-} + R^{+} - R^{0}_{T1}$

Method	Basis set	C1	H2	C3	H4	C5	H6	C7	H8	C1+H2	C3+H4	C5+H6	C7+H8
CASPT2, 0-IPEA	6-31G(d,p)	0.0019	-0.0001	-0.0016	-0.0001	0.0018	-0.0001	-0.0016	-0.0001	0.0018	-0.0017	0.0017	-0.0017
CASPT2, 0-IPEA	cc-pVDZ	0.0014	0.0000	-0.0012	-0.0001	0.0013	0.0000	-0.0014	-0.0001	0.0014	-0.0013	0.0013	-0.0015
CASPT2, 0-IPEA	cc-pVTZ	-0.0012	0.0003	0.0006	0.0003	-0.0012	0.0003	0.0006	0.0003	-0.0009	0.0009	-0.0009	0.0009
CASPT2, S-IPEA	6-31G(d,p)	-0.0001	-0.0001	0.0003	-0.0001	-0.0002	-0.0001	0.0003	-0.0001	-0.0002	0.0002	-0.0003	0.0002
CASPT2, S-IPEA	cc-pVDZ	0.0004	0.0000	-0.0002	0.0000	0.0003	0.0000	-0.0002	0.0000	0.0004	-0.0002	0.0003	-0.0002
CASPT2, S-IPEA	cc-pVTZ	-0.0004	0.0004	-0.0003	0.0004	-0.0005	0.0003	-0.0003	0.0003	0.0000	0.0001	-0.0002	0.0000
CASSCF	6-31G(d,p)	0.0001	0.0000	0.0000	0.0000	0.0001	0.0000	0.0000	0.0000	0.0001	0.0000	0.0001	0.0000
CASSCF	cc-pVDZ	0.0001	-0.0001	0.0001	-0.0001	0.0001	-0.0001	0.0001	-0.0001	0.0000	0.0000	0.0000	0.0000
CASSCF	cc-pVTZ	-0.0005	0.0004	-0.0003	0.0004	-0.0005	0.0004	-0.0003	0.0004	-0.0001	0.0001	-0.0001	0.0001
CASSCF	cc-pVQZ	-0.0028	0.0027	-0.0026	0.0027	-0.0028	0.0027	-0.0026	0.0027	-0.0001	0.0001	-0.0001	0.0001
DFT(B3LYP)	6-31G(d,p)	0.0004	-0.0004	0.0004	-0.0004	0.0004	-0.0004	0.0004	-0.0004	0.0000	0.0000	0.0000	0.0000
DFT(B3LYP)	cc-pVDZ	0.0006	-0.0006	0.0006	-0.0006	0.0006	-0.0006	0.0006	-0.0006	0.0000	0.0000	0.0000	0.0000
DFT(B3LYP)	cc-pVTZ	0.0009	-0.0009	0.0009	-0.0009	0.0009	-0.0009	0.0009	-0.0009	0.0000	0.0000	0.0000	0.0000
DFT(B3LYP)	cc-pVQZ	0.0003	-0.0003	0.0004	-0.0003	0.0003	-0.0003	0.0003	-0.0003	0.0000	0.0000	0.0000	0.0000
DFT(M06-2X)	6-31G(d,p)	-0.0005	0.0005	-0.0005	0.0005	-0.0006	0.0005	-0.0005	0.0005	0.0000	0.0000	0.0000	0.0000
DFT(M06-2X)	cc-pVDZ	0.0002	-0.0002	0.0002	-0.0002	0.0001	-0.0002	0.0002	-0.0002	0.0000	0.0000	0.0000	0.0000
DFT(M06-2X)	cc-pVTZ	0.0050	-0.0050	0.0050	-0.0050	0.0050	-0.0050	0.0050	-0.0050	0.0000	0.0000	0.0000	0.0000
DFT(M06-2X)	cc-pVQZ	0.0039	-0.0039	0.0040	-0.0039	0.0039	-0.0039	0.0039	-0.0039	0.0000	0.0000	0.0000	0.0000
DFT(CAM-B3LYP)	6-31G(d,p)	0.0005	-0.0005	0.0004	-0.0005	0.0005	-0.0005	0.0005	-0.0005	0.0000	0.0000	0.0000	0.0000
DFT(CAM-B3LYP)	cc-pVDZ	0.0007	-0.0006	0.0007	-0.0006	0.0006	-0.0006	0.0006	-0.0006	0.0000	0.0000	0.0000	0.0000
DFT(CAM-B3LYP)	cc-pVTZ	0.0012	-0.0011	0.0011	-0.0011	0.0011	-0.0011	0.0011	-0.0011	0.0001	0.0000	0.0000	-0.0001
DFT(CAM-B3LYP)	cc-pVQZ	0.0169	-0.0168	0.0168	-0.0168	0.0168	-0.0168	0.0168	-0.0168	0.0000	0.0000	0.0000	0.0000

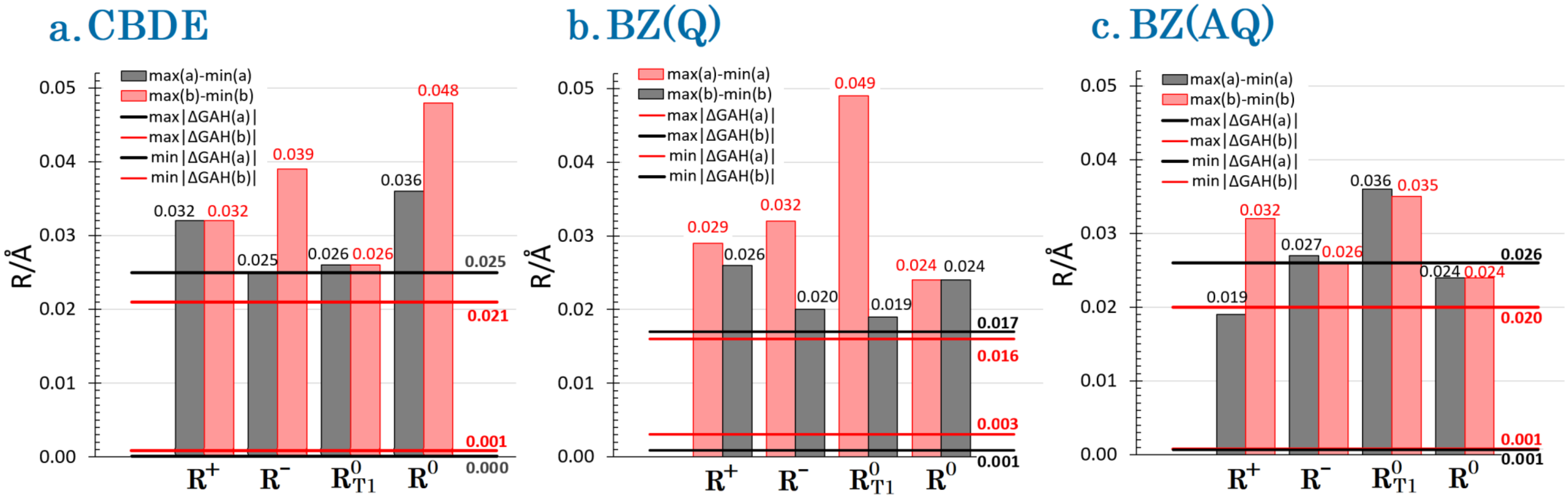

Figure S1. Minimum (**min|ΔGAH(R)|**) and maximum (**max|ΔGAH(R)|**) of unsigned values of **Eq. 1** expression on the background of statistic ranges **max(R)-min(R)** of **a** and **b** bonds lengths of quinoid (**Q**) and anti-quinoid (**AQ**) benzene (**BZ**) conformers and for cyclobutadiene (**CBDE**) for all investigated computational approaches, and all basis sets applied. Data for shorter bond are marked as red of pink, whereas data for longer ones are black or grey (see **Fig. 1**). For more detailed data see **Tables S1-S14**.
